# Integrated systems immunology approach identifies impaired effector T cell memory responses as a feature of progression to severe dengue fever

**DOI:** 10.1186/s12929-023-00916-4

**Published:** 2023-04-13

**Authors:** Lisa J. Ioannidis, Stephanie I. Studniberg, Emily M. Eriksson, Suhendro Suwarto, Dionisius Denis, Yang Liao, Wei Shi, Alexandra L. Garnham, R. Tedjo Sasmono, Diana S. Hansen

**Affiliations:** 1 The Walter and Eliza Hall Institute of Medical Research, Parkville, VIC Australia; 2 Department of Medical Biology, The University of Melbourne, Parkville, VIC Australia; 3 Division of Tropical and Infectious Diseases, Department of Internal Medicine, Faculty of Medicine, Universitas Indonesia, Cipto Mangunkusumo National Hospital (RSCM), Jakarta, Indonesia; 4 Eijkman Research Center for Molecular Biology, Jakarta, Indonesia; 5 Olivia Newton-John Cancer Research Institute, Heidelberg, VIC Australia; 6 School of Mathematics and Statistics, The University of Melbourne, Parkville, VIC Australia; 7 Department of Microbiology, Monash Biomedicine Discovery Institute, Monash University, Clayton, VIC Australia

**Keywords:** Dengue fever, Dengue hemorrhagic fever, Effector memory T cells, Non-classical monocytes

## Abstract

**Background:**

Typical symptoms of uncomplicated dengue fever (DF) include headache, muscle pains, rash, cough, and vomiting. A proportion of cases progress to severe dengue hemorrhagic fever (DHF), associated with increased vascular permeability, thrombocytopenia, and hemorrhages. Progression to severe dengue is difficult to diagnose at the onset of fever, which complicates patient triage, posing a socio-economic burden on health systems.

**Methods:**

To identify parameters associated with protection and susceptibility to DHF, we pursued a systems immunology approach integrating plasma chemokine profiling, high-dimensional mass cytometry and peripheral blood mononuclear cell (PBMC) transcriptomic analysis at the onset of fever in a prospective study conducted in Indonesia.

**Results:**

After a secondary infection, progression to uncomplicated dengue featured transcriptional profiles associated with increased cell proliferation and metabolism, and an expansion of ICOS^+^CD4^+^ and CD8^+^ effector memory T cells. These responses were virtually absent in cases progressing to severe DHF, that instead mounted an innate-like response, characterised by inflammatory transcriptional profiles, high circulating levels of inflammatory chemokines and with high frequencies of CD4^low^ non-classical monocytes predicting increased odds of severe disease.

**Conclusions:**

Our results suggests that effector memory T cell activation might play an important role ameliorating severe disease symptoms during a secondary dengue infection, and in the absence of that response, a strong innate inflammatory response is required to control viral replication. Our research also identified discrete cell populations predicting increased odds of severe disease, with potential diagnostic value.

**Supplementary Information:**

The online version contains supplementary material available at 10.1186/s12929-023-00916-4.

## Background

Dengue is an infection caused by one of four dengue viruses in the family *Flaviviridae*. The virus is transmitted by infected *Aedes aegypti* and *Aedes albopictus*, which are day-biting urban mosquitoes that feed mainly on humans. These vectors are widespread throughout tropical and sub-tropical regions, leaving 70% of the world population at risk of dengue. The disease burden is estimated to 390 million clinical cases per year [1], 500,000 of which are severe enough to require hospitalization. Around 75% of the global population exposed to dengue is in the Asia-Pacific region [2], and the economic cost due to dengue-associated disease in this region has been estimated at US$ 2.36 billion, with a burden of disease as disability-adjusted life years (DALY) of 214,000 (~ 372 DALYs per million inhabitants) [3, 4].

Dengue virus (DENV) is a positive-sense single-stranded RNA virus, that encodes a polyprotein with three structural proteins and seven nonstructural proteins. The envelope protein (E protein) is involved in receptor binding, membrane fusion, and viral assembly. E protein is the main target of neutralizing antibodies and contains the four DENV serotype-specific antigenic epitopes (DENV-1 to 4). Monocytes, macrophages and dendritic cells in the spleen, lymph nodes, lungs, liver, kidney, and stomach are the primary cellular hosts of DENV in humans [5, 6].

Dengue fever (DF) typically starts from four to seven days after a person is bitten by an infected mosquito. Disease manifests as a sudden onset of fever (up to 40 ºC) that lasts up to ten days and is accompanied by headache and muscle pain in the back and limbs. Other symptoms of moderate dengue include nausea, vomiting, pain in the eyes and rashes on the upper and lower limbs. A proportion of cases can progress to severe dengue also known as dengue hemorrhagic fever (DHF), which is associated with increased vascular permeability leading to thrombocytopenia and hemorrhages. If left untreated this could become life-threatening resulting in severe plasma leakage leading to dengue shock syndrome (DSS), characterized by fluid accumulation causing respiratory distress, severe bleeding and multi-organ failure. There is no specific treatment for dengue, and care is mainly supportive. Oral rehydration and analgesia are routinely used. Intravenous fluids, blood transfusions and intensive care are the required courses of treatment for severe cases. To date, at first presentation, there is no validated way of identifying which individuals will progress to severe manifestations of the disease [7, 8], and in endemic areas, patients are often admitted for inpatient observation, as doctors tend to err on the side of caution. It is estimated that the cost of a non-fatal hospitalized dengue case is US $1394 [3]. Thus, strategies to predict disease progression will not only improve patient management but will also have a substantial socio-economic impact on health systems.

The adaptive immune response plays a key role in protection against DENV, with both T cell-mediated and antibody responses contributing to the control of viral load. Neutralizing antibodies are an important immune defense mechanism against the virus. While primary dengue infection induces a serotype-specific long-lasting neutralizing antibody response able to protect from re-infection [9], cross-reactive antibodies targeted against the other DENV serotypes only protect for a few months [10]. During secondary heterotypic infections, non-neutralizing cross-reactive antibodies from the first infection bind to virions of the second serotype to form DENV-antibody complexes, which are more readily taken up by Fc-gamma-receptor (FcγR)-bearing myeloid cells than uncoated virus particles [11]. This process, known as antibody dependent enhancement (ADE), results in higher levels of viral progeny and has been proposed to lead to severe dengue [12], explaining why secondary DENV infections are usually at higher risk of DHF compared to primary infections.

Similar to antibodies, T cell responses also appear to play a dual role in dengue [13]. CD8^+^ T cells have been shown to protect against secondary DENV infection in mice and humans [14, 15] and contribute to the antiviral response by killing infected cells and secreting IFN-γ. CD4^+^ T cells contribute to the control of infection by facilitating B cell and CD8^+^ T cell activation and secreting cytokines [16]. On the other hand, it has been suggested that CD8^+^ T cells against a primary DENV serotype can dominate the response to secondary heterotypic infection, resulting in excessive cytokine responses that can worsen disease and induce pathology [17, 18]. It has been also suggested that in heterologous DENV infection, weak affinity cross-reactive CD8^+^ T cells expand and compete with protective T cells, resulting in inefficient control of the virus and immune pathology [19]. Moreover, DENV-specific CD4^+^ T cells also produce higher amounts of TNF, a cytokine contributing to disease in DHF patients, in response to heterotypic antigen [20]. To date, the specific phenotypes of T cells associated with protection and susceptibility to severe dengue have not been fully defined.

The fact that antibody-mediated and T cell-mediated responses appear to participate in both protection from infection and dengue pathogenesis has complicated the identification of reliable correlates of immunity, which poses a caveat in the evaluation of effective anti-DENV vaccines. These considerations have also deterred the identification of cellular parameters or biomarkers to predict the clinical course of disease. To address these issues, we pursued a systems biology approach integrating plasma chemokine profiling, high-dimensional mass cytometry and peripheral blood mononuclear cell (PBMC) transcriptional profiling in a prospective study of individuals from a dengue-endemic area that progressed to develop either DF or DHF. High frequencies of CD4^low^ non-classical monocytes were associated with progression to DHF and predicted increased odds of severe disease at the onset of fever. Progression to severe dengue was also associated with a transcriptional signature featuring impaired T cell activation and reduced cell metabolism compared to individuals with mild disease manifestation. In contrast, uncomplicated dengue cases featured an expansion of ICOS^high^CD4^+^ and CD8^+^ effector memory T cells. Our research suggests that efficient T cell activation might play a critical role in attenuating severe disease symptoms during secondary DENV infections. The results also provide proof of concept for the potential of systems biology approaches to identify immunological signatures predicting increased odds of DHF to develop diagnostic tools for early detection of complicated cases.

## Methods

### Study population

A prospective, observational study of dengue cases was conducted at Pondok Indah Hospital in Jakarta, Indonesia. A dengue positive diagnosis was confirmed by detection of DENV NS1 antigen at point of care (SD Bioline, Korea). RT-PCR to determine DENV serotype was done using the Simplexa Dengue Real-time RT-PCR Kit (DiaSorin, Salugglia, Italy). The four DENV serotypes were found among participants, with DENV-2 being the most prevalent (58%), followed by DENV-4 (18%) and DENV-1/-3 (12% each). Consenting participants with a confirmed diagnosis (aged between 16 and 64 years) donated a venous blood sample at first presentation (day 1–3 of fever), and plasma and PBMCs were frozen. Virus-specific IgM and IgG antibodies were measured in plasma samples collected at first presentation using Panbio Dengue Duo IgM and IgG Capture ELISA (Alere), to classify cases between primary and secondary infections based on the IgM/IgG ratio. Participants with a positive IgM and negative IgG result were classified as primary infections, while secondary infections were identified by a positive IgG which could be accompanied by positive IgM result. Participants were monitored daily for disease progression (DF or DHF) for 10 days as previously described [21]. Based on routine laboratory tests and clinical evaluation, the attending physicians classified the dengue disease grade based on 2011 WHO guidelines [22]. Briefly, cases with clinical symptoms, including headache, retro-orbital or abdominal pain, vomiting, fluid accumulation, evidence of skin or mucosal bleed, liver enlargement and an increase in haematocrit with a concurrent rapid reduction of platelet count (below 10 × 10^3^/ml) were classified as DHF.

### Luminex assay for chemokine assessment

Chemokines from uninfected healthy controls and confirmed dengue cases were quantified in plasma samples by Luminex^®^ (R&D systems) assay. Magnetic beads were formulated into pre-designed microparticle cocktails with capture antibodies against human CCL2, CCL17, CXCL5, CXCL8, CXCL9, CXCL10 and CXCL11. Dilutions for reagents, standards, and samples were performed following the manufacturer’s protocol. Diluted samples, standards, and microparticle cocktails were added to microplate wells and incubated for 2 h at 22 ºC with agitation. After washing three times with washing buffer, a biotin-antibody cocktail was added, and plates were incubated for an additional hour at 22 ºC. After three washes, samples were incubated in the presence of a streptavidin-PE conjugate for 30 min. After a final wash, plates were read using a Luminex 100/200 instrument with xPONENT software (Luminex Corp., Austin, TX, USA). Mean fluorescence intensity values were used to estimate chemokine concentration, extrapolating from standard curves.

### Cytometry by time-of-flight

PBMCs (1–2 × 10^6^) from DF and DHF cases as well as non-infected dengue-naive Melbourne controls were stained with 5 µM Cell-ID Cisplatin (Fluidigm) in PBS for 5 min at room temperature. Cells were then blocked with Human TruStain FcX (Biolegend) and stained with a cocktail of surface marker antibodies (Additional file 1: Table S1) in CyTOF staining buffer (PBS with 0.5% bovine serum albumin [BSA; Sigma] and 0.02% sodium azide [Sigma]) for 30 min at room temperature. After surface staining, cells were washed twice with CyTOF staining buffer and then incubated with 125 nM Cell-ID iridium intercalator (Fluidigm) in Maxpar fix and perm buffer (Fluidigm) for a minimum of 18 h. Prior to data acquisition, cells were washed twice by centrifugation in ultrapure water and then resuspended in a 1/10 dilution of 4-Element EQ normalization beads (Fluidigm) in ultrapure water. Cells were analysed on a Helios model mass cytometer (Fluidigm) at ~ 300 events/s. Data were normalized using the signal from 4-Element EQ Beads (Fluidigm) as previously described [23]. Manual gating was then performed using FlowJo version 10 (BD Biosciences) to exclude doublets and dead cells, before individual cell populations were selected and exported for further analysis in Cytobank [24]. Individual cell populations were then visualized using viSNE [25], while FlowSOM [26] was used to identify cell sub-populations. Self-organizing maps (SOMs) were generated for each cell population using hierarchical consensus clustering on the tSNE axes. The parameters included in each viSNE analysis are shown in Additional file 2: Table S2. CITRUS [27] was used to identify differentially abundant cell populations using the same parameters as the viSNE analysis at a 1% false-discovery rate (FDR).

### RNA-sequencing

RNA was extracted from 2 × 10^5^ PBMCs from selected samples with sufficient material to allow assessment of multiple endpoints using the RNeasy Plus Mini Kit (QIAGEN) following manufacturers’ instructions. RNA was quantified with RNA Screen Tape on the Agilent TapeStation 2200 System. Libraries were prepared with either 50 ng or 25 ng of total RNA using the Illumina TruSeq RNA Library Prep Kit (< 100 ng) v1.0 following manufacturers’ instructions and submitted for sequencing by paired end, 80 bp reads on an Illumina NextSeq 500 platform.

### Transcriptional analysis

Raw sequence reads in FASTQ file format were aligned to the human reference genome GRCh38/hg38 using the align() function in Rsubread version 2.10.5 with default parameters [28, 29]. Fragments of aligned sequences overlapping Gencode human genes (GRCh38.p13) were quantified with featureCounts [30], with Gencode version 38 primary assembly annotation used in the quantification. Genes with no symbols, sex-linked genes, immunoglobulin genes, and non-protein-coding genes were filtered out from the analysis. Haemoglobin genes were found to be highly variable and were also filtered from the analysis. Genes with low counts in fewer than 5 samples were determined as unexpressed and filtered out using the filterByExpr function in edgeR version 3.38.4 [31], leaving 13,013 genes for differential expression analysis. Filtered counts were normalised using the trimmed mean of M-values method [32] in edgeR. A design matrix was constructed incorporating the group, and adjusting for day from fever onset as follows:

$$\mathrm{design} \leftarrow \mathrm{model.matrix ( 0 + group + feverday)}.$$

Counts were transformed to log2-CPM (logCPM), precision weighted and quantile normalised using the voomwithQualityWeights() function to down-weight within-group variability [33, 34] in limma version 3.52.2 [35]. A linear model was fitted to each gene and differential expression was assessed using robust empirical Bayes moderated *t*-statistics [36] with a cut-off of 5% applied for calling differentially expressed genes. Entrez Gene IDs for differentially expressed genes were entered into the goana() and kegga() functions [37] in limma to determine over-representation of differentially expressed genes in Gene Ontology (GO) terms. Lists of differentially expressed genes between pairwise comparisons were also entered into the Ingenuity Pathway Analysis software platform (QIAGEN Inc., https://www.qiagenbioinformatics.com/products/ingenuity-pathway-analysis) for canonical pathway and upstream regulator analysis. Differential enrichment of functional immune pathways was determined using the tmodLimmaTest() function in the tmod package version 0.46.2 [38] with blood transcription modules [39] and hallmark gene signatures from the molecular signatures database (mSigDB) [40, 41] as gene sets. Significant GO pathways were visualised using the GOPlot package version 1.0.2 [42]. Heatmaps were generated using the pheatmap (version 1.0.12) and ComplexHeatmap (version 2.12.1) [43] packages, and chord diagrams were generated using the circlize package version 0.4.15 [44] Rotational gene set testing and barcode plots were generated using the limma package in R [35].

### Statistical analysis

Characteristics of clinical groups were compared using unpaired t-tests or ANOVA for continuous data that was normally distributed, while Mann–Whitney tests or Kruskal Wallis tests were used for data that did not follow normal distribution. Chi-squared tests were used to evaluate nominal data. The false discovery rate was controlled to below 5% using the method of Benjamini and Hochberg [45] throughout the study. Correlations were determined using Spearman’s rank correlation as indicated and visualised using the network_plot() function in the corrr package version 0.4.4. Logistic regression models were fitted between groups to determine the odds ratio for cell populations. Hierarchical clustering heatmaps were calculated using the complete method and Euclidian distance matrix. Statistical analyses were performed in GraphPad prism version 9 and R version 4.2.0.

## Results

### Cohort characteristics

**Fig. 1:**
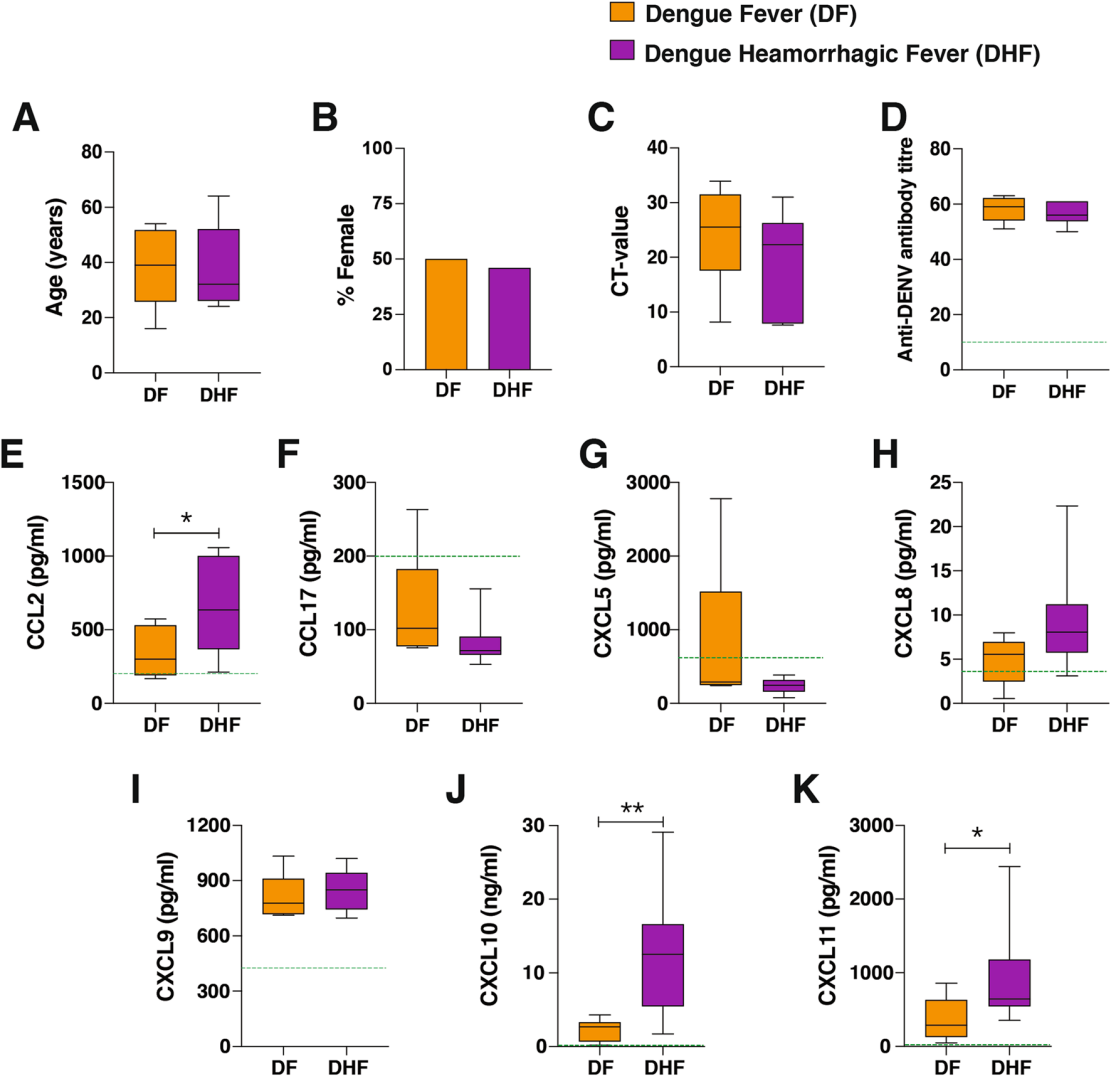
Study cohort characteristics. Individuals with a confirmed secondary DENV infection were recruited for the study at the onset of fever and followed up for progression to DF (n = 7) or DHF (n = 10). **A–D**. Clinical parameters determined in the study include age (**A**), gender (**B**) and CT-value (**C**), and anti-DENV IgG antibody titres (**D**). Boxes represent the 25th to 75th percentiles, whiskers show the range (minimum to maximum), and lines represent the median of biological replicates. The dotted line depicts the average antibody background levels of uninfected healthy controls (n = 5). Line plots depict mean ± SD. Significance was determined by the Kruskal–Wallis test (**A**), the Chi-square test (**B**), and the Mann-Whitney test (**C**, **D**), *p < 0.05. **E–K**. Mean chemokine levels. CCL2 (**E**), CCL17 (**F**), CXCL5 (**G**), CXCL8 (**H**), CXCL9 (**I**), CXCL10 (**J**) and CXCL11 (**K**) were determined in plasma samples of study participants. Boxes represent the 25th to 75th percentiles, whiskers show the range (minimum to maximum), and lines represent the median of 7 (DF) and 10 (DHF) biological replicates. The dotted line depicts the average antibody background levels of uninfected healthy controls (n = 5). Significance was determined by the Mann-Whitney test. *p < 0.05, **p < 0.01

The clinical characteristics of the study participants are summarised in Fig. 1. The study recruited individuals with a confirmed dengue positive diagnosis. Study participants with a confirmed secondary DENV infection were selected for this analysis. Consenting participants donated a venous blood sample at enrolment (day 1–3 of fever) and were then monitored daily for disease progression to DF or DHF for 10 days as described in Methods. There was no difference in age or gender composition between study participants (Fig. 1A, B) and viral loads were similar between clinical groups (Fig. 1C). Assessment of antibody responses to the DENV envelope protein at first presentation revealed that all DF and DHF cases had high virus-specific IgG titres (Fig. 1D). Plasma chemokine levels were also assessed at first presentation. CCL17, CXCL5 and CXCL8 levels in both clinical groups were similar to background levels in healthy controls. While CXCL9 levels were comparable in DF and DHF cases, CCL2, CXCL10 and CXCL11 levels were increased in DHF cases compared with the DF group (Fig. 1E–K). Thus, all individuals in this arm of the study have a confirmed secondary DENV infection, and individuals progressing to severe illness produced high levels of inflammatory chemokines at the onset of fever.

### High-dimensional mass cytometry identifies lymphocyte sub-populations associated with different dengue disease outcomes

**Fig. 2:**
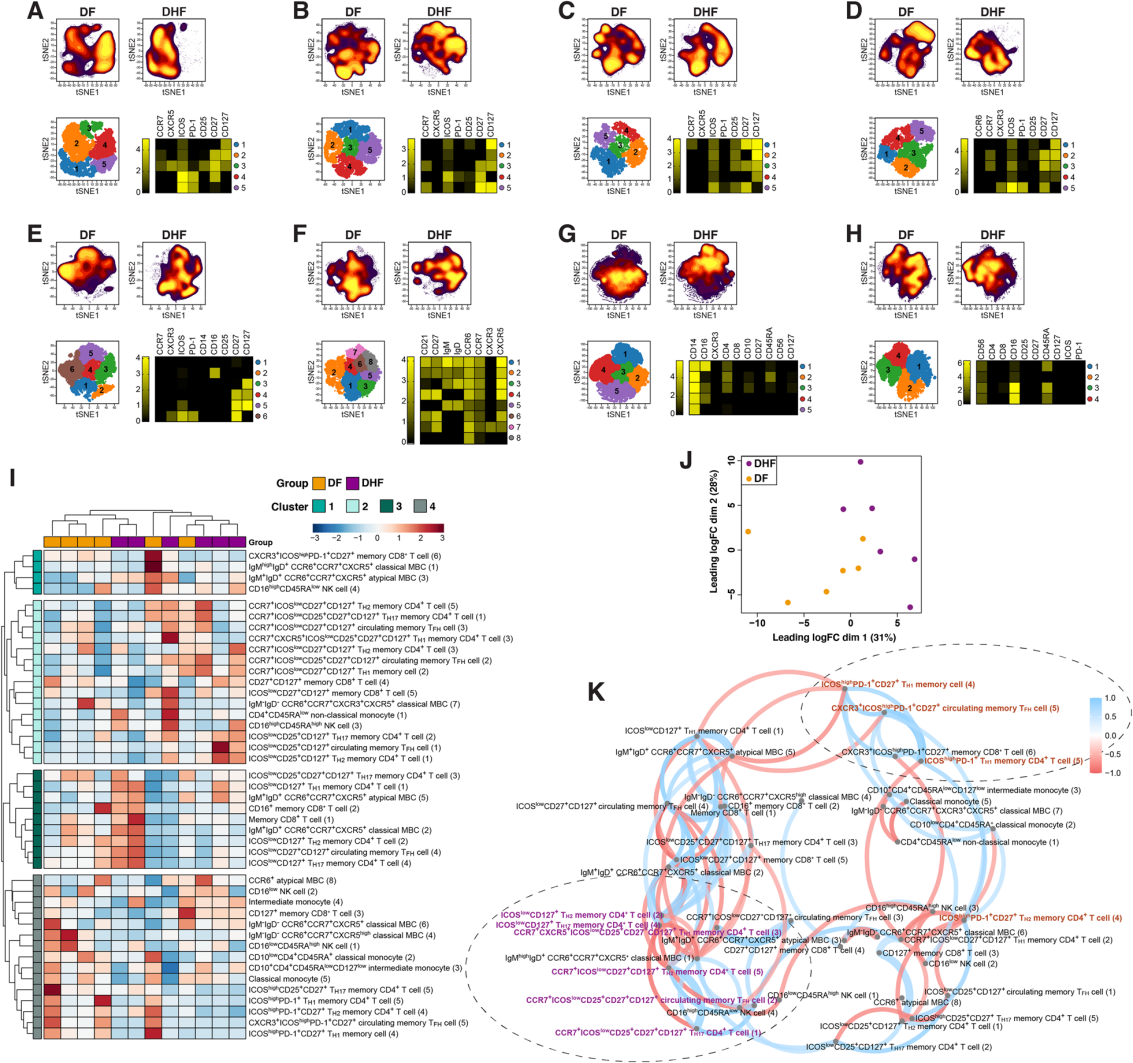
Identification of memory CD4^+^ and CD8^+^ T cells, MBCs, monocyte and NK cell sub-populations in DENV-infected individuals. **A–K** PBMCs collected at first presentation from DENV-positive individuals progressing to DF (n = 6) or DHF (n = 6) were stained with a panel of metal-labelled antibodies and analysed by CyTOF. tSNE analysis was performed and FlowSOM clustering was used to identify individual cell sub-populations within gated: **A** T_H1_ memory CD4^+^ T cells (CD19^−^CD3^+^CD4^+^CD45RA^−^CCR6^−^CXCR3^+^), **B** T_H2_ memory CD4^+^ T cells (CD19^−^CD3^+^CD4^+^CD45RA^−^CCR6^−^CXCR3^−^), **C** T_H17_ memory CD4^+^ T cells (CD19^−^CD3^+^CD4^+^CD45RA^−^CCR6^+^CXCR3^−^), **D** Circulating memory T_FH_ cells (CD19^−^CD3^+^CD4^+^CD45RA^−^CXCR5^+^), **E** Memory CD8^+^ T cells (CD19^−^CD3^+^CD8^+^CD45RA^−^). **F** MBCs (CD3^−^CD19^+^CD20^+^CD10^−^), **G** Monocytes (CD3^−^CD19^−^CD14^+^), **H**. NK cells (CD3^−^CD19^−^CD14^−^CD56^+^). The tSNE plots in the top panel display cell density and represent the pooled data for each group, while the lower panel shows a projection of the FlowSOM clusters on a tSNE plot. Heatmaps show the median marker expression for each FlowSOM cluster. **I** Unsupervised hierarchical clustering heatmap showing the frequency of all FlowSOM clusters across all clinical samples. **J** Unsupervised multi-dimensional scaling of all populations identified by FlowSOM clustering across all clinical samples. **K** Spearman correlation networks were used to examine the relationship between cell populations identified by FlowSOM clustering induced in response to infection. Blue lines represent positive correlations, and red lines represent negative correlations. The distance between variables is determined by multidimensional scaling and represents the strength of the correlation, where highly correlated variables are positioned closer together

To identify lymphocyte sub-populations associated with progression to DF or DHF after a secondary DENV infection, PBMCs of study participants collected at the onset of fever were stained with a panel of metal-labelled antibodies against a range of B cell, T cell, Natural Killer (NK) cell and monocyte markers (Additional file 1: Table S1) and analysed by cytometry by time of flight (CyTOF). Within the circulating memory CD4^+^ T cell compartment, surface expression of the chemokine receptors CCR6 and CXCR3 allows the identification of T helper (T_H_)_1_-like (CCR6^−^CXCR3^+^), T_H2_-like (CCR6^−^CXCR3^−^) and T_H17_-like (CCR6^+^CXCR3^−^) cells, while circulating memory T follicular helper (T_FH_) cells can be identified by expression of CXCR5 (Additional file 3: Fig. S1). To explore the composition of these memory CD4^+^ T cell populations, tSNE analysis and FlowSOM clustering were performed, and marker expression was assessed in each cell population. This approach allowed for the identification of five distinct sub-populations of T_H1_ memory CD4^+^ T cells, T_H2_ memory CD4^+^ T cells, T_H17_ memory CD4^+^ T cells and memory T_FH_ cells expressing variable levels of chemokine receptors and surface markers including CCR7, ICOS, PD-1, CD25, CD27 and CD127 (Fig. 2A–D). A similar approach was used to explore the composition of the memory CD8^+^ T cell pool, with six distinct sub-populations identified (Fig. 2E).

Within the circulating memory B cell (MBC) pool, CD21 and CD27 expression can be used to identify classical MBCs (CD27^+^CD21^+^) and atypical MBCs (CD27^−^CD21^−^). tSNE analysis and FlowSOM clustering of the CD19^+^CD20^+^CD10^−^ MBC pool facilitated the identification of five classical and three atypical MBC sub-populations, including populations of both IgM^+^IgD^+^ and class-switched (IgM^−^IgD^−^) cells expressing variable levels of the chemokine receptors CCR6, CCR7, CXCR3 and CXCR5 (Fig. 2F), present in the blood of dengue patients at first presentation. Within the innate cell compartment, FlowSOM clustering of gated CD14^+^ monocytes revealed two classical (CD14^+^CD16^−^), two intermediate (CD14^+^CD16^low^) and one non-classical (CD14^+^CD16^high^) monocyte sub-population (Fig. 2G) in the blood of dengue patients. Four sub-populations expressing different levels of CD16 were also identified among CD3^−^CD56^+^ NK cells (Fig. 2H).

The frequency of each cell sub-population identified by FlowSOM clustering was determined. Unsupervised hierarchical clustering (Fig. 2I) and multi-dimensional scaling (Fig. 2J) of all sub-populations segregated most DF and DHF cases. Four main groups of cell populations were identified by unsupervised hierarchical clustering, with trends towards cell populations more abundant in cases progressing to DHF in clusters 1 and 2, and cell populations preferentially expressed among individuals progressing to DF in clusters 3 and 4 (Fig. 2I). Interestingly, cluster 4 included classical monocytes as well as sub-populations of memory T_FH_ cells, T_H1_ and T_H2_-polarised CD4^+^ T cells expressing high levels of inducible T cell costimulatory (ICOS), programmed cell death protein 1 (PD-1) and CD27. Spearman correlation analysis indicated that frequencies of these T cell populations (marked in brown in Fig. 2K) were highly correlated with each other (Fig. 2K). In contrast, subsets of memory T_FH_ cells, T_H1_, T_H2_ and T_H17_ polarised CD4^+^ T cells in clusters 1–2 (marked in purple in Fig. 2K), apparently enriched among individuals progressing to DHF were also correlated with each other and were characterized by high expression levels of CCR7 and CD127.

**Fig. 3:**
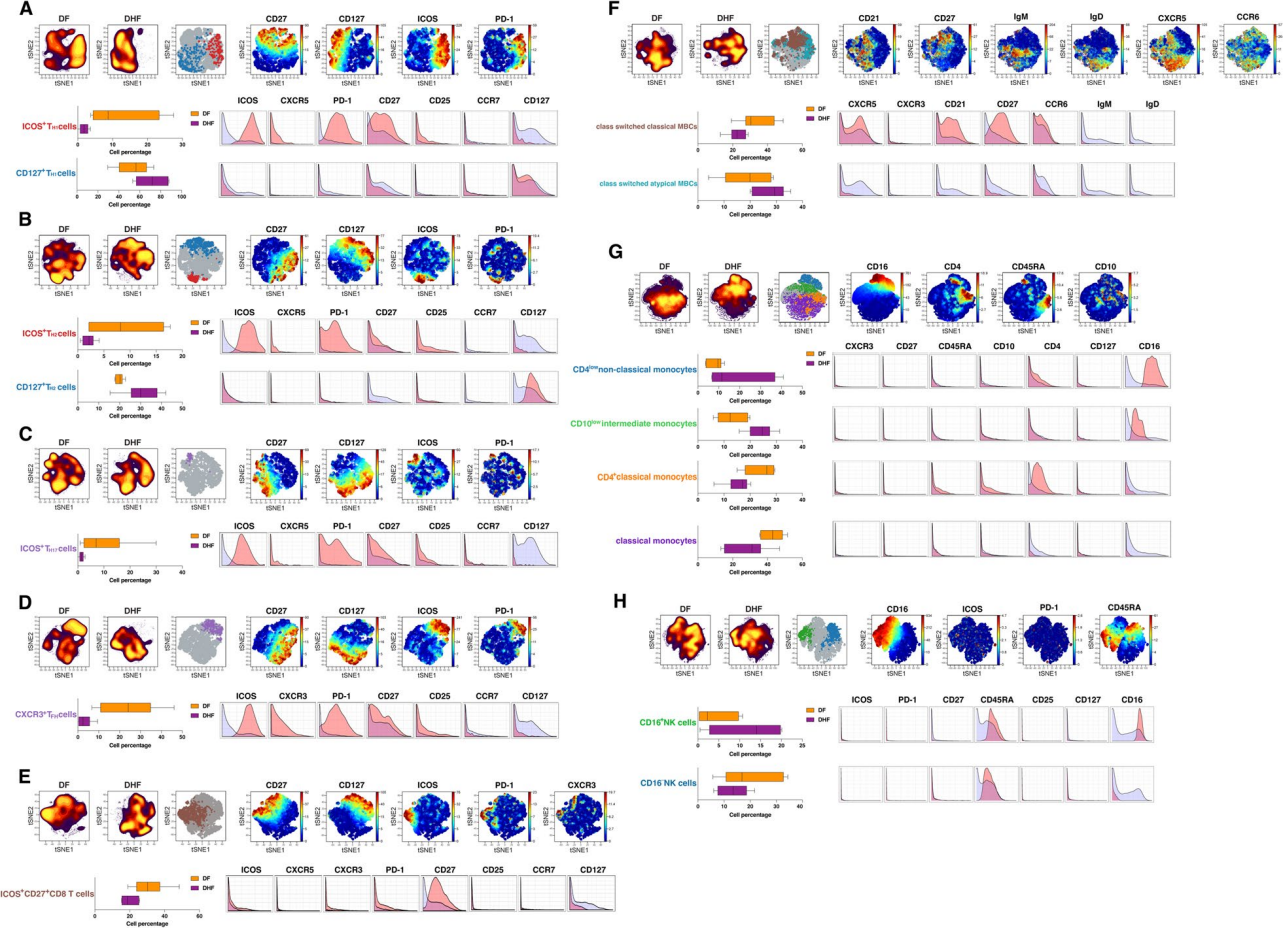
Subsets of T cells, MBCs, monocytes and NK cells associated with progression to different dengue fever outcomes. **A–H**. Unsupervised identification of differentially abundant cell populations between DF (n = 6) or DHF (n = 6) clinical groups was performed using CITRUS (FDR < 1%). Differentially abundant populations were identified among: (**A**) T_H1_ memory CD4^+^ T cells; (**B**) T_H2_ memory CD4^+^ T cells; (**C**) T_H17_ memory CD4^+^ T cells; (**D**) circulating memory T_FH_ cells; (**E**) memory CD8^+^ T cells; (**F**) MBCs; (**G**) monocytes; (**H**) NK cells. The tSNE plots in the top of each panel display cell density and represent pooled data for each clinical group as calculated in the clustering analysis shown in Fig. 2A-G, while the middle panels show differentially abundant populations identified in colours on a tSNE overlay, and the viSNE plots on the left-hand side from each top panel depict relevant marker expression on tSNE overlays. The lower left panels show the frequency of differentially abundant cell populations identified by CITRUS. Boxes represent the 25th to 75th percentiles, whiskers show the range (minimum to maximum), and lines represent the median of 6 (DF) and 6 (DHF) biological replicates. The lower right panels illustrate marker expression in differentially abundant populations (pink histograms), relative to background expression (lilac histograms)

To further explore these responses, unsupervised identification of differentially abundant cell populations between clinical groups was performed using the CITRUS algorithm [27] (FDR < 1%). This analysis identified two populations of memory T_H1_-polarised CD4^+^ T cells differentially abundant between clinical groups. ICOS^+^PD-1^+^CD27^+^CCR7^−^ cells were virtually absent in DHF cases. These cells, resembling an effector memory phenotype, were highly abundant in individuals that progressed to DF. In contrast, CD127^+^ cells were significantly higher in DHF cases relative to DF counterparts (Fig. 3A). A highly similar pattern was observed in the memory T_H2_ CD4^+^ T cell pool, with a subset of ICOS^+^PD-1^+^CD27^+^CCR7^−^ cells significantly higher in DF compared to DHF cases and CD127^+^ cells enriched in DHF individuals compared to those with mild disease (Fig. 3B). Furthermore, the same population of ICOS^+^PD-1^+^CD27^+^CCR7^−^ cells was also identified as significantly higher in DF compared to DHF cases in the memory T_H17_-CD4^+^ T cell compartment (Fig. 3C). CITRUS analysis also identified one population of CXCR3^+^ memory T_FH_ cells and an additional population of CXCR3^+^ memory CD8^+^ T cells significantly more abundant in DF compared to DHF cases, also displaying high levels of ICOS, PD-1, and CD27 (Fig. 3D, E). Thus, together these results suggest that during a secondary infection, progression to uncomplicated dengue is associated with the expansion of CD4^+^ and CD8^+^ T cells consistent with an effector memory phenotype and expressing high levels of co-stimulatory molecules.

CITRUS analysis revealed two populations of MBCs differentially abundant between clinical groups. Whereas CXCR5^+^CCR6^+^ class-switched classical MBCs were higher in DF individuals, class-switched atypical MBCs were significantly enriched in DHF cases compared to DF counterparts (Fig. 3F). In the monocyte compartment, classical monocytes and CD4^+^ classical monocytes were significantly higher in DF compared to DHF cases, while a population of CD4^low^ non-classical monocytes and a population of CD10^low^ intermediate monocytes were higher in individuals that developed DHF (Fig. 3G). Moreover, highly abundant in patients progressing to DHF was a subset of CD16^+^ NK cells, whereas their CD16^−^ counterparts were significantly higher in DF cases (Fig. 3H). Importantly, downstream analysis of the 15 populations identified as differentially abundant by CITRUS using manual gating, showed that except for classical MBCs, CD4^+^ classical monocytes (which were significantly reduced in DHF cases), and CD16^+^ NK cells, blood frequencies of all cell types were negligible among dengue-naive healthy controls compared to dengue-exposed study participants (Additional file 3: Fig. S2), confirming the expansion of these cellular compartments in response to DENV infections progressing to different disease outcomes. Thus, whereas CD16^+^ NK cells and non-classical monocytes appear to be associated with progression to severe dengue, T_H1_-polarised T_FH_ cells and ICOS^high^ CD4^+^ and CD8^+^ effector memory T cells are features associated with more favourable infection outcomes after a secondary DENV infection.

### RNA sequencing of PBMCs at the onset of fever segregates transcriptional profiles of individuals progressing to DF or DHF

**Fig. 4:**
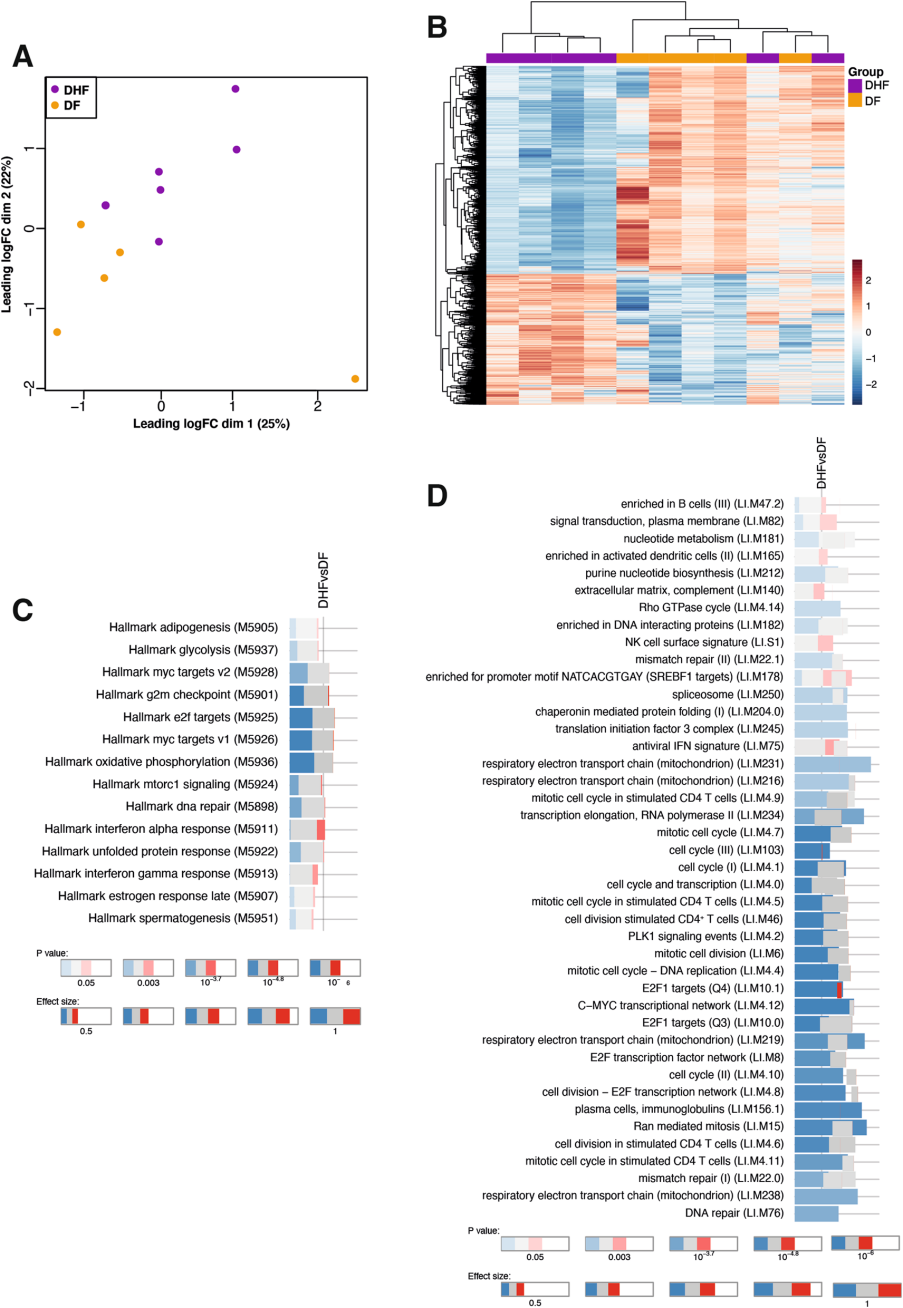
RNA-seq of PBMCs segregates transcriptional profiles of individuals progressing to DF or DHF. **A–D** PBMCs collected at first presentation from DENV-positive individuals progressing to DF (n = 5) or DHF (n = 6) were selected for RNA-seq analysis. **A** Unsupervised multi-dimensional scaling of the top 500 most variably expressed genes across all samples. **B** Unsupervised hierarchical clustering of the 2411 differentially expressed genes between DF and DHF cases using the complete method and Euclidian measure of distance. **C–D** tmod gene set enrichment analysis showing significant BTMs differentially enriched for pairwise comparisons (FDR < 5%).

To identify molecular pathways associated with progression to DF or DHF, selected study participants with sufficient material were chosen for transcriptional profiling by RNA-sequencing (RNA-seq). Multi-dimensional scaling and unsupervised hierarchical clustering of transcriptional profiles discriminated between individuals progressing to DF and DHF (Fig. 4A, B). A design matrix was created with clinical groups as a factor with adjustment for day of fever upon hospital presentation. The design matrix was then incorporated into linear modelling and gene expression estimation for the identification of differentially expressed genes between DHF and DF. A total of 2411 genes were found to be differentially expressed, with 929 upregulated and 1482 downregulated in patients progressing to DHF compared with patients progressing to DF (Fig. 4B). To identify immunological processes underlying differences in transcriptional profiles, differential enrichment was assessed with the gene set enrichment tool tmod [38], using pairwise comparisons between clinical groups and blood transcriptional modules (BTMs) [39] as gene sets (Fig. 4C, D). Modules involved in type-I interferons, apoptosis and an NK cell signature were significantly enriched in DHF compared to DF cases. Conversely, modules involved in cell division, cell cycle, glycolysis, oxidative phosphorylation as well as plasma cells were significantly underrepresented in cases progressing to severe DHF compared to DF counterparts.

### Progression to DHF features transcriptional profiles supporting innate inflammatory responses, reduced cell division and reduced oxidative metabolism relative to DF

**Fig. 5:**
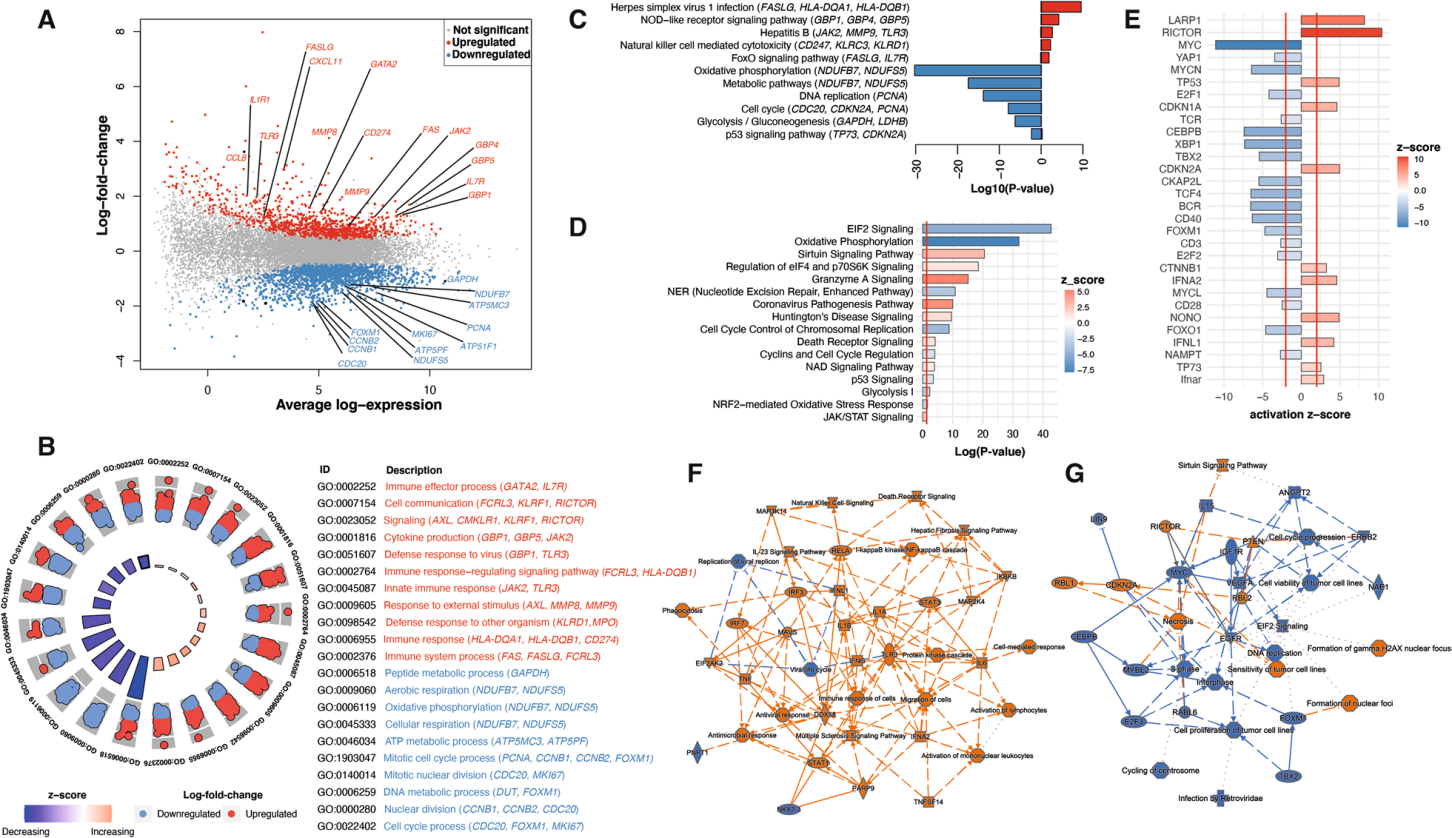
Transcriptional profiles supporting inflammatory processes and reduced cell proliferation and metabolism in individuals progressing to DHF. **A–G** Gene expression profiles of PBMCs from DENV-infected individuals progressing to DF (n = 5) or DHF (n = 6) were compared. **A** Mean-difference plot displaying genes differentially expressed between DF and DHF cases. Each gene is plotted as a single point determined by log-fold-change and average transcript abundance. Red genes are overrepresented, and blue genes are underrepresented in DHF relative to DF. **B** Plots showing significantly enriched GO terms scaled by Log10(P-value). Red GO terms are upregulated and blue GO terms are downregulated in DHF compared to DF. **C** Bar plots showing significantly enriched KEGG pathways scaled by Log10(P-value). Red KEGG pathways are upregulated and blue KEGG pathways are downregulated in DHF compared to DF. **D** IPA canonical pathways significantly overrepresented in differentially expressed genes between DF and DHF cases scaled by Log10(P-value). Pathways with a positive z-score in red are activated in DHF relative to DF, and pathways with a negative z-score in blue are inhibited in DHF compared to DF. The red line corresponds to a P value of 0.05. **E** Upstream regulator analysis of the 2411 differentially expressed genes between DHF and DF. The red lines represent a significant activation z-score of ±2. **F–G** Graphical summaries depicting networks of genes and downstream processes activated in cases progressing to DHF relative to DF (**F**) or processes enriched in patients progressing to DF relative to DHF (**G**)

To further define transcriptional signatures preferentially activated by individuals progressing to DF or DHF, various platforms were pursued for gene set enrichment analysis (Fig. 5A–D). Gene ontology (GO) terms (Fig. 5B), Kyoto Encyclopedia of Genes and Genomes (KEGG) pathways (Fig. 5C), and Ingenuity Pathway Analysis (IPA) (Fig. 5D) showed significant upregulation of pathways involved in innate immune response to microorganisms in individuals progressing to DHF. This included genes encoding several cytokine/chemokines and cytokine/chemokine receptors such as *IL1R1, IL7R, CCL8* and *CXCL11*, signalling molecules and transcription factors governing the stress response such as *JAK2* and *GATA2*, various guanylate-binding proteins (*GBP1, GBP4, GBP5*), all IFN-γ-inducible proteins known to exert anti-microbial activity, along with *TLR3*, a toll like receptor responsible for recognition of double stranded RNA. Furthermore, genes encoding protein products involved in apoptosis, such as *FAS, FASLG* and *CD274* (also known as programmed death-ligand 1), and metalloproteinases (*MMP8, MMP9*), upregulated in various pathogenic conditions, were also among the genes upregulated in patients progressing to DHF.

Upstream regulator analysis identified increased activation of rapamycin-insensitive companion of mTOR (*RICTOR*) and La-related protein 1 (*LARP1*) pathways in DHF compared to DF patients (Fig. 5E). Both pathways are critical in the control of mTOR signalling that governs cell proliferation and metabolism and have been shown to be modulated by viral infections to support viral replication [46–48]. The transcription regulator Non-POU domain-containing octamer-binding protein [49] as well as the cytokine IFN-alpha-2 (*IFNA2*) and its receptor *IFNAR* were also upstream regulators in patients progressing to DHF, with several genes involved in stress response and inflammatory processes predicted as targets in these type I IFN-mediated pathways (Fig. 5E). A graphical summary, used to identify the main entities featured by transcriptional profiles of DHF cases predicts that *TLR3* activation (recognising viral RNA) activates this inflammatory cytokine response, resulting mainly in NK cell and monocyte activation, a strong antimicrobial response and downstream inhibition of viral replication (Fig. 5F).

Two main signature pathways appeared to be downregulated in individuals progressing to DHF and enriched in patients with milder disease manifestations: cell proliferation and cell metabolic processes including, oxidative phosphorylation, cellular respiration and glycolysis. These included genes encoding cyclins such as *CCNB1* and *CCNB2*, cell activation marker *MKI67*, replication factors such as *PCNA* and *CDC20*, as well as transcriptional regulator *FOXM1* (Fig. 5A–D). Genes encoding several enzymes critical for cell metabolic processes such as *GAPDH, NDUFB7, NDUFS5* as well as *ATP5MC3*, *ATP51F1* and *ATP5PF* required for ATP metabolism, were also reduced in DHF compared to DF patients. Upstream regulator and graphical summary analysis predicted key transcription factors involved in the control of cell division such as *MYC, FOXM1* and *FOXO1*, as well as T cell activation molecules (*CD3, CD40*, and *CD28*) as key elements responsible for these transcriptional signatures, suggesting that these pathways could become preferentially activated in individuals who progressed to mild DF (Fig. 5D, G).

To address this, and to specifically confirm that these pathways modulated before the onset of DF or DHF are enriched or diminished relative to homeostatic transcriptional levels of healthy uninfected controls, we compared our list of 2411 differentially expressed genes with a previously described transcriptional signature of 1303 genes differentially expressed upon unclassified DENV infection relative to uninfected controls [50]. This analysis identified a significant enrichment of 393 genes in common between both data sets (Additional file 3: Fig. S3A–C). Notably, gene set enrichment of these 393 genes using GO terms, KEGG pathways, and IPA showed identical transcriptional signatures compared to the results in Fig. 5, with an innate-like anti-viral stress response featuring upregulation of TNF and type-1 interferons before progression to DHF, and a significant enrichment of terms involved in cell proliferation, cell metabolism, and T cell activation in individuals that did not develop severe hemorrhagic symptoms. Thus, together these results suggest that after a secondary DENV infection, progression to DHF is associated with an innate immune transcriptional signature as well as impaired activation and expansion of the T cell compartment.

Since other studies have previously analysed transcriptional profiles of cases progressing to DF or DHF in other locations, we sought to determine if our results were aligned with findings obtained in different settings. To that end, we identified two multi-cohort analyses [51, 52] that together integrated 12 unique gene expression datasets [53–64] capturing 466 [51] and 365 [52] clinical samples of patients progressing to DF or DHF (Additional file 3: Fig. S4A). Whereas the first multi-cohort analysis framework discovered a 20-gene set predictive of severe dengue, the second one identified an 8-gene predictive signature. Importantly, the accuracy of these signatures was validated in independent prospective cohort studies. When these two gene signatures were combined and compared with our list of 2411 differentially expressed genes, rotational gene set testing using the ROAST test in limma [35] revealed a significant enrichment (p < 0.01) of 10 genes from the 28-gene signature (Additional file 3: Fig. S4B, C, D). Gene set enrichment analysis of these shared genes using GO terms showed transcriptional profiles featuring innate and antimicrobial humoral immunity (Additional file 3: Fig. S4E). Thus, despite the limitations of a small sample size, our findings appear to be significantly aligned with validated gene signatures predicting progression to DHF.

### Uncomplicated dengue fever transcriptional profiles correlate with effector memory T cell responses to DENV infection

**Fig. 6:**
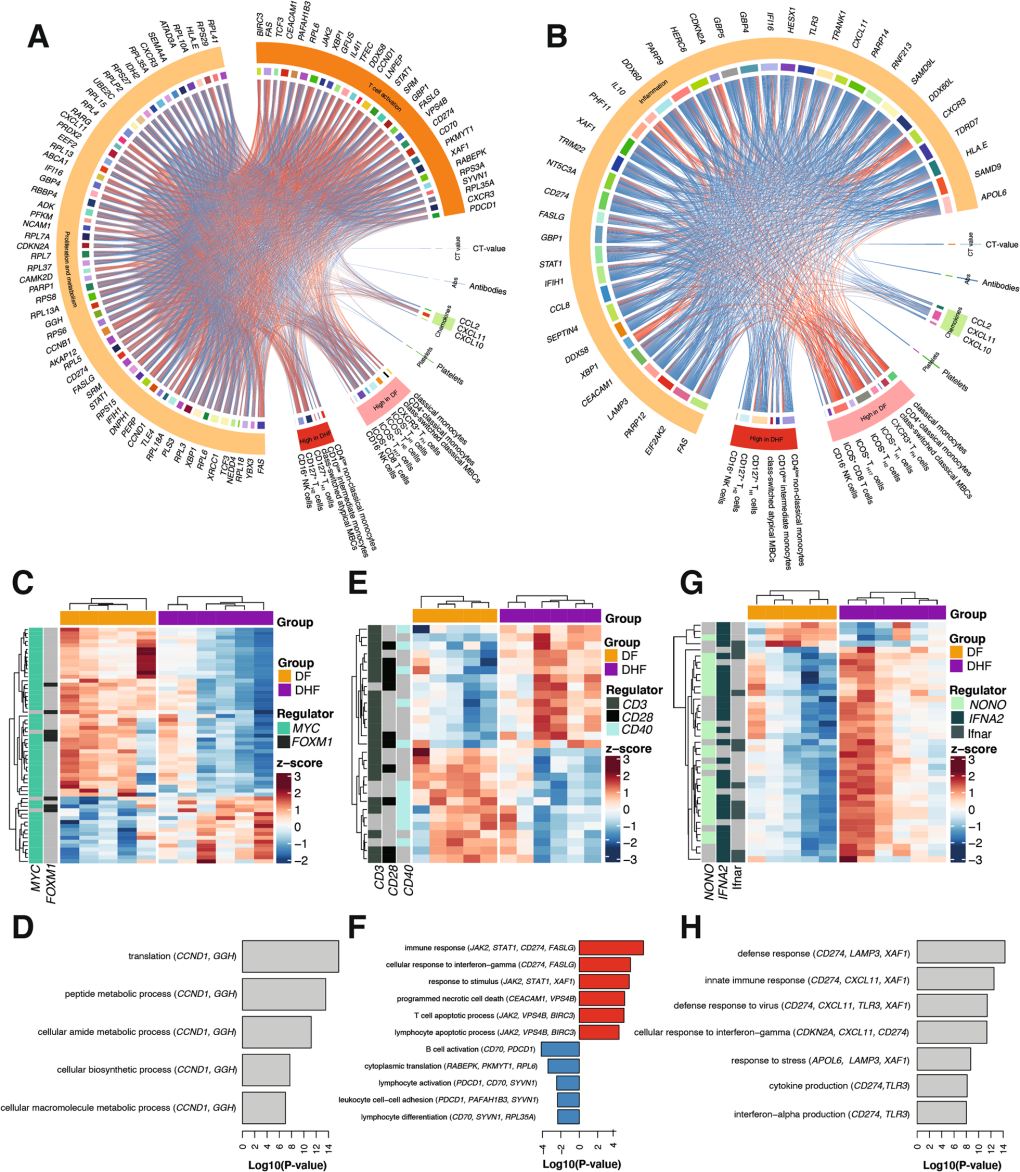
Integrative analysis of cellular signatures and transcriptional profiles in cases progressing to DF or DHF. **A** Chord diagram integrating associations between DF signature genes, immune cell populations and chemokine responses. Blue lines within the chord diagram represent positive correlations between two variables, while red lines represent negative correlations. (Benjamini-Hochberg adjusted Spearman’s Rho, FDR < 5%). **B** Chord diagram integrating associations between DHF signature genes, immune cell populations and chemokine responses. Blue lines within the chord diagram represent positive correlations between two variables, while red lines represent negative correlations. (Benjamini-Hochberg adjusted Spearman’s Rho, FDR < 5%). **C** Hierarchical clustering heatmap of genes predicted to be under the control of upstream regulators *MYC* and *FOXM1* amongst patients progressing to DF or DHF. **D** Bar plots showing significantly enriched GO terms scaled by Log10(P-value) for genes in heatmap in C. **E** Hierarchical clustering heatmap of genes predicted to be under the control of upstream regulators *CD3, CD28* and *CD40* amongst patients progressing to DF or DHF. **F** Bar plots showing significantly enriched GO terms scaled by Log10(P-value) for genes in heatmap in E. Red GO terms are upregulated and blue GO terms are downregulated in DHF compared with DF. **G** Hierarchical clustering heatmap of genes predicted to be under the control of upstream regulators *NONO, IFNA2* and *IFNAR* amongst patients progressing to DF or DHF. **H** Bar plots showing significantly enriched GO terms scaled by Log10(P-value) for genes in heatmap in G

To integrate immune responses associated with progression to DF or DHF identified by CyTOF and blood transcriptional signatures, Spearman correlations (Benjamini-Hochberg adjusted FDR < 5%) were applied (Fig. 6A, B). For this analysis, genes identified to be under the control of signature upstream regulators identified in Fig. 5 by IPA analysis were used. In general, cell populations overrepresented in individuals progressing to DF were positively correlated with the proliferation/metabolism and T cell activation transcriptional signatures, with ICOS^+^ memory T_H1_ and T_H17_ cells, as well as CXCR3^+^ T_FH_ cells featuring the highest number of associations with gene expression profiles (Fig. 6A). Hierarchical clustering of these transcriptional signatures confirmed that most of the differentially expressed genes in the data set predicted to be under the control of upstream regulators *MYC* and *FOXM1* were upregulated in cases progressing to DF compared to DHF and featured GO terms specifically associated with cellular metabolic processes (Fig. 6C, D). Nearly half of the genes predicted to be under the control of upstream regulators *CD3, CD28* and *CD40* were upregulated in DF relative to DHF cases (Fig. 6E). Gene set enrichment analysis revealed that whereas terms involved in lymphocyte activation were upregulated in DF cases, genes encoding protein products supporting apoptosis and IFN-γ responses were upregulated in cases progressing to DHF (Fig. 6F).

In general, the inflammatory transcriptional signature featured by cases progressing to DHF was negatively correlated with cell clusters abundant in DF cases, but showed positive associations with cell populations overrepresented in DHF cases, with CD4^low^ non-classical monocytes and memory CD127^+^ T_H1_ cells showing the highest number of correlations (Fig. 6B). The 10-gene set shared between our differentially expressed gene list and multi-cohort defined gene signatures [51, 52] also showed positive correlations with atypical MBCs, abundant among DHF cases (Additional file 3: Fig. S5). Almost all these genes, under the control of upstream regulators *NONO, IFNA2* and *IFNAR* and featuring GO terms involved in antiviral and inflammatory cytokine response, were clearly upregulated in individuals progressing to DHF compared to DF counterparts. Thus, together these findings suggest that the expansion of the effector memory CD4^+^ and CD8^+^ T cell pool observed in DF cases (Figs. 2, 3) might be supported by increased metabolic rates, required to induce activation and proliferation of this compartment, and in the absence of this adaptive response, a type-I innate response is required to control the infection, which is associated with progression to severe disease manifestations.

### The balance between classical and non-classical circulating monocytes predicts risk of DHF after primary and secondary DENV infection

Figures 2, 3, 4, 5 and 6 suggest that in the absence of robust effector memory T cell responses in DHF cases, a innate immune response, including activation of NK cells and monocytes is the main defence against the virus during a secondary DENV infection. Therefore, we sought to determine (1) whether a similar response pattern was observed in cases progressing to DHF after a primary DENV infection, and (2) if defined innate cell populations in the blood could be good predictors of odds of severe hemorrhagic illness at the onset of fever. To that end, PBMCs collected at first presentation from participants that had a confirmed primary DENV infection (high IgM and no detectable IgG virus-specific antibodies) were stained with a panel of metal-labelled antibodies against monocyte and NK cell markers (Additional file 1: Table S1) and analysed by CyTOF.

The clinical characteristics of the primary infection arm of the study were similar to those with secondary infection and are summarized in Additional file 3: Fig. S6. There was no difference in age or gender composition between study participants (Additional file 3: Fig. S6B), with a trend towards higher viral loads that did not reach significance in DHF cases (Additional file 3: Fig. S6C). Unlike the secondary infection arm of the cohort that identified high levels of some chemokines in cases progressing to DHF, no significant differences were found in plasma CCL17, CXCL5, CXCL8 and CXCL9, CCL2, CXCL10 and CXCL9 levels between clinical groups (Additional file 3: Fig. S6D–J).

**Fig. 7:**
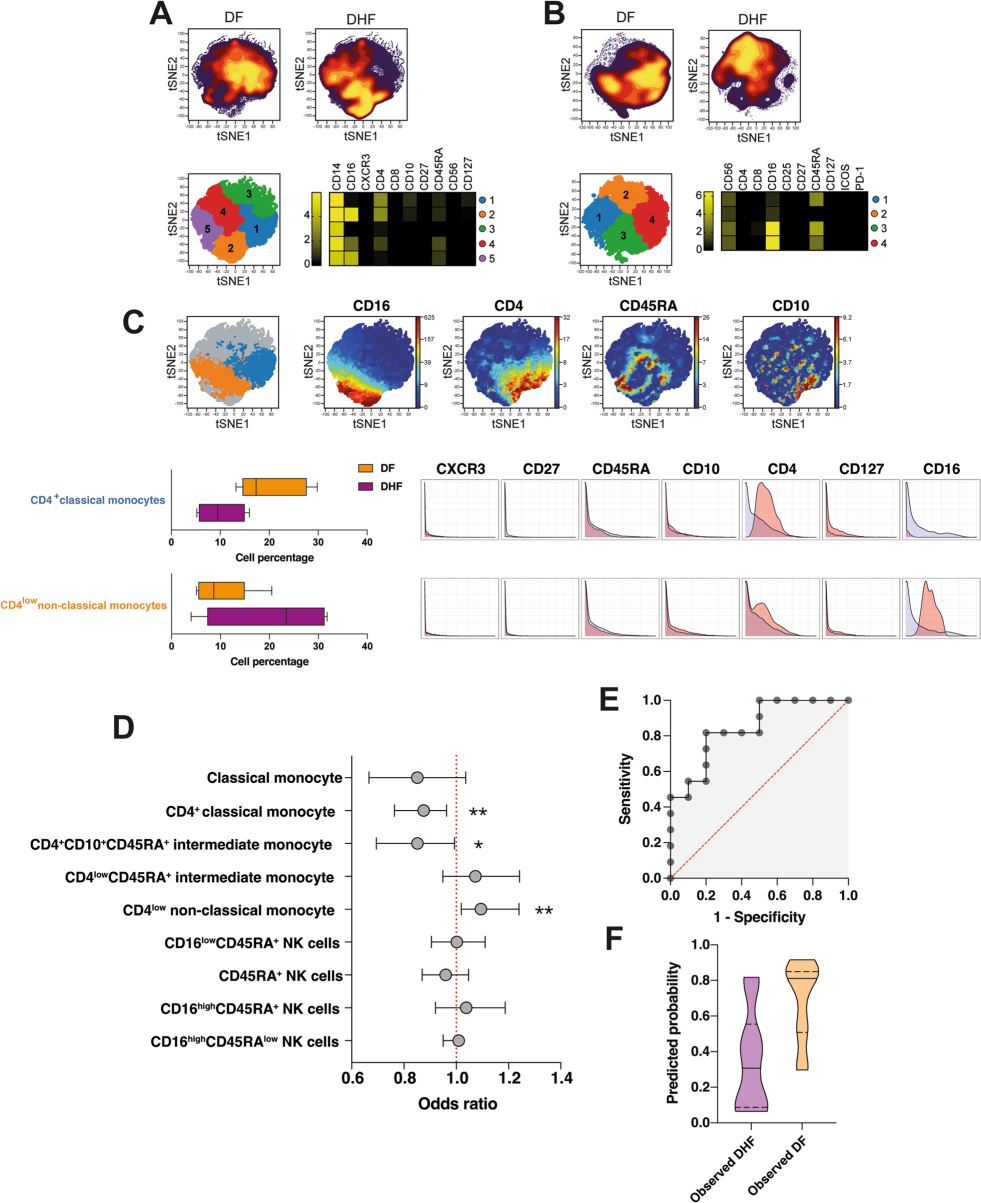
High frequencies of circulating CD4^low^ non-classical monocytes predict increased odds of DHF. **A–C **PBMCs collected at first presentation from DENV-positive individuals progressing to DF (n = 5) or DHF (n = 4) after a primary infection were stained with a panel of metal-labelled antibodies and analysed by CyTOF. tSNE analysis was performed and FlowSOM clustering was used to identify individual cell sub-populations within gated CD3^−^CD19^−^CD14^+^ monocytes (**A**) and CD3^−^CD19^−^CD14^−^CD56^+^ NK cells (**B**). The tSNE plots in top panels display cell density and represent the pooled data for each group, while the lower panel shows a projection of the FlowSOM clusters on a tSNE plot. Heatmaps show the median marker expression for each FlowSOM cluster (**C**). Differentially abundant populations were identified by CITRUS among gated monocytes. The top panels show differentially abundant populations identified in colours on a tSNE overlay, and the viSNE plots on the right-hand side from each top panel depict relevant marker expression on tSNE overlays. The lower left panels show the frequency of differentially abundant cell populations identified by CITRUS. Boxes represent the 25th to 75th percentiles, whiskers show the range (minimum to maximum), and lines represent the median of 6 (DF) and 6 (DHF) biological replicates. The lower right panels illustrate marker expression in differentially abundant populations (pink histograms), relative to background expression (lilac histograms). **D** Odds ratios as determined by logistic regression showing associations between cell frequencies and the risk of progressing to DHF. Symbols represent the odds ratio estimated using 10 patients progressing to DHF, and 11 patients progressing to DF after primary or secondary infection. The vertical lines depict the 95% confidence interval. *p < 0.05, **p < 0.01. **E–F** ROC curve (**E**) and predicted probability plot (**F**) classifying individuals progressing to DF (n = 11) or DHF (n = 10) at first presentation based on the frequency of CD4^+^ classical monocytes and CD4^low^ non-classical monocytes

To explore the composition of innate cells after primary DENV infection, tSNE analysis and FlowSOM clustering were performed within gated monocytes (Fig. 7A) as well as CD3^−^CD56^+^ NK cells (Fig. 7B), and marker expression was assessed in each cell population. Similar to our findings after secondary infection, this analysis revealed two classical (CD14^+^CD16^−^), two intermediate (CD14^+^CD16^low^) and one non-classical (CD14^+^CD16^high^) monocyte sub-population (Fig. 7A) in the blood of primary dengue cases. Four subsets of NK cells, expressing different levels of CD16 were also identified by FlowSOM clustering (Fig. 7B). The CITRUS algorithm was then applied for unsupervised identification of differentially abundant cell populations between clinical groups (FDR < 1%). Whereas no differentially abundant populations of NK cells were found, similar to secondary infection, CD4^+^ classical monocytes were significantly higher in individuals developing DF compared to cases progressing to DHF, and CD4^low^ non-classical monocytes were highly abundant in cases that progressed to DHF (Fig. 7C).

Next, all samples from the primary and secondary arms of the study were combined, and tSNE and FlowSOM analysis performed to obtain frequencies of all monocyte and NK cell sub-populations across the entire cohort (Additional file 3: Fig. S7). To define associations between cell populations and risk of developing DHF, logistic regression models were applied (Benjamini-Hochberg adjusted FDR < 5%). Whereas high frequencies of CD4^+^ classical and CD4^+^CD10^+^CD45RA^+^ intermediate monocytes were associated with reduced odds of DHF, high frequencies of CD4^low^ non-classical monocytes predicted increased odds of progressing to DHF at the onset of fever (Fig. 7D). The frequencies of these cell populations were then assessed for their ability to classify individuals progressing to DF or DHF. Figure 7E F show the ROC curve and predicted frequencies of the best performing model. Circulating levels of CD4^+^ classical and CD4^low^ non-classical monocytes were able to correctly classify individuals developing either DF or DHF with a high degree of accuracy (AUROC 0.8455, p = 0.0075), providing proof of concept for the potential of defined monocyte populations in the blood identified by single cell approaches to predict odds of severe dengue at the onset of fever.

## Discussion

Secondary DENV infections are usually more severe than a primary exposure to the virus and are a well-accepted risk factor for DHF and DSS [65]. A large body of data supports the concept that exposure to DENV results in the development of protective as well as disease-enhancing adaptive immune responses upon re-infection, with both antibody-mediated and T cell responses contributing to these processes [9, 11, 16–18, 66]. To untangle the complexity of this immunological landscape, this study pursued a systems biology approach integrating clinical parameters, plasma chemokine levels, high-dimensional mass cytometry, and blood transcriptional profiling in adults from a dengue-endemic area in Indonesia to identify cellular and molecular processes associated with progression to DF or DHF after a second exposure to DENV. We found that whereas progression to uncomplicated disease manifestations was associated with increased cell proliferation and metabolism transcriptional profiles and an expansion of CD4^+^ and CD8^+^ effector memory T cells, progression to severe DHF featured a type-I immune response characterised by inflammatory transcriptional profiles as well as high circulating levels of inflammatory chemokines and non-classical monocytes. Thus, our results are consistent with a model in which effector memory T cell activation could play an important role to ameliorate severe disease during a secondary DENV infection, and in the absence of these processes, a strong innate response is required to control viral replication, at the expense of producing an inflammatory environment that contributes to the induction of clinical symptoms.

Over the past decade, numerous studies investigated transcriptional profiles of dengue cases to identify markers of disease progression, with varied results. Similar to our findings here, progression to severe dengue has been shown to be often [52, 53, 57, 59, 67–69] but not always [54, 58, 61, 70] associated with transcriptional signatures featuring NK cell activation, increased apoptosis, upregulation of type I interferon and IFN-γ-mediated pathways. In contrast to this innate-like inflammatory transcriptional response associated with progression to DHF, our bioinformatic analysis identified T cell activation molecules and master transcription factors such as *MYC*, *FOXM1* and *FOXO1*, known to support cell proliferation and govern a range of metabolic processes, as regulators of transcriptional profiles in patients progressing to uncomplicated disease manifestations. These gene expression profiles were tightly correlated with frequencies of ICOS^+^ effector memory T cells and CXCR3^+^ T_FH_ cells, lending support to the idea that the increased metabolic transcriptional signature observed had developed to support the energy demands of a proliferating T cell pool, which was expanding upon a secondary encounter with the virus.

Elegant longitudinal studies demonstrated that both effector memory CD4^+^ and CD8^+^ T cells are induced after exposure to DENV and that these cells are long-lived, persisting for 12 months post-infection [71–73]. Consistent with those findings, our high-dimensional single-cell cytometry analysis identified various populations of ICOS^+^PD-1^+^CD27^+^CCR7^−^ T_H1_, T_H2_ and T_H17_-polarised CD4^+^ T cells as well as CD8^+^ T cells with an effector memory phenotype only 2–3 days after the onset of fever, suggesting the prior presence and rapid expansion of these cells upon a secondary DENV infection. Notably, these cells were only present in individuals progressing to mild disease manifestations and virtually absent in patients that developed DHF. Similarly, transcriptional profiles of asymptomatic DENV-infected individuals [62] featured increased T cell activation relative to clinical dengue counterparts, and multi-functional CD4^+^ and CD8^+^ T cells have been found at higher frequencies in outpatients compared to hospitalized dengue cases [74]. Together, this evidence supports the concept that adaptive T cell immunity, and particularly effector memory T cells, might play a critical role in attenuating disease severity during a secondary DENV infection and associate with good infection outcomes. In our study, all effector memory CD4^+^ and CD8^+^ T cell subsets found in individuals progressing to uncomplicated dengue expressed high level of co-stimulatory molecules, including ICOS. Expression of this co-stimulatory molecule is usually found in various T cell types, including T regulatory cells, T_FH_ cells and effector T cells. In these cells, ICOS plays an important role in cell activation, maintenance, and survival [75]. Emerging research is highlighting the importance of ICOS co-stimulation to deliver efficient anti-tumour effector T cell responses [76–78], with various ICOS agonist antibodies currently in clinical trial showing promising results [79, 80]. Targeting this co-stimulatory pathway could be also beneficial to boost adaptive immunity and improve disease outcomes in secondary dengue infections.

Unsupervised analysis of the B cell compartment identified a subpopulation of CD21^−^CD27^−^ atypical MBCs preferentially expanded in DHF cases. These cells have been detected in other viral [81] and parasitic [82] infections and share features in common with a subset called age-associated B cells [83]. In these settings, atypical MBCs have been found to have low differentiating capacity into antibody-secreting cells upon re-encounter with antigen and similar to our results here, associations with poor disease outcomes [83, 84, 85]. In contrast, class-switched classical MBCs dominated the response in individuals progressing to DF. Although anti-DENV antibody titres in the study were comparable across individuals with different infection outcomes, our results do not exclude the possibility that antibodies with different neutralising capacity and/or affinity for antigen could arise after stimulation and differentiation of these MBC subsets present in DF or DHF cases and play a role in the progression towards different clinical outcomes.

CXCR5^+^CD4^+^ circulating memory T_FH_ cells have been shown to provide help to B cells and share many functional features with T_FH_ cells in the lymphoid organs [86]. These cells have been previously found to become activated in response to DENV and to correlate with plasmablast frequencies [87, 88]. Similar to conventional T helper cells, the expression of the lineage-defining chemokine receptors CXCR3 and CCR6 allows the differentiation of T_H1_-, T_H2_- and T_H17_-polarised memory T_FH_ cells, and previous findings suggested that CXCR3 expression in T_FH_ cells is associated with their functional capacity. Although it was initially proposed that CXCR3^+^ cells have reduced helper capacity compared to CXCR3^−^ cells [89], CXCR3^+^ T_FH_ cells have been also found to succesfully expand and secrete cytokines, particularly in response to various viral infections [90, 91]. Consistent with these findings, our analysis here identified a population of CXCR3^+^ T_FH_ cells correlating with classical MBCs and only present in study participants with mild disease manifestations. Further work will be required to determine if CXCR3^+^ T_FH_ cells provide help to support the production of efficient neutralising antibody responses that protect against severe dengue disease symptoms.

Monocytes are one of the main targets of DENV and several studies have assessed the relative abundance of classical, intermediate and non-classical subsets during acute infection [56, 92–94]. How the composition of this compartment fluctuates in patients with different disease severity in DENV infections is less understood. Here, using unsupervised high-dimensional data analysis, we found that whereas populations of classical monocytes are abundant at the onset of fever in individuals developing uncomplicated dengue, non-classical monocytes dominate the blood response in patients progressing to DHF, suggesting that these cells could contribute to the induction of severe clinical symptoms. In support to this idea, non-classical monocytes have been shown to upregulate expression of adhesion molecules and chemokine receptors that facilitate endothelial cell adhesion [94] in response to acute DENV infection. These cells are also major producers of pro-inflammatory cytokines including TNF, IL-1β, and IL-6 [95]. TNF is a potent activator of the vascular endothelium known to modulate the permeability of endothelial cells [96], raising the possibility that local production of TNF by non-classical monocytes could contribute to vascular leakage in DHF cases. Non-classical monocytes may also promote ADE by supporting the differentiation of B cells into plasmablasts [56] that produce short-lived, low affinity antibodies and have been identified as one of the main cellular targets of DENV in the blood that becomes activated in severe cases [68].

The currently used warning signs to identify dengue cases at risk of developing severe ilness are based on clinical parameters that appear late in the disease and are not always specific. This situation promotes ineffective patient triage and resource allocation [7, 8]. Recent studies estimated that the total cost per hospitalized dengue case raises to US$1,250 [97] in Indonesia, with the total economic burden associated to hospitalized dengue cases estimated to US$355.2 million [97]. Thus, strategies to predict the burden of disease will have a substantial socio-economic impact in dengue endemic regions. From all parameters induced in response to DENV identified by our systems biology approach, the balance between defined populations of classical and non-classical monocytes in the blood was the best predictor of odds of DHF after 1–3 days of fever. Furthermore, unlike inflammatory chemokines, CD4^low^ non-classical monocytes were detectable in cases progressing to DHF, not only after secondary but also primary DENV infections, in which other risk factors such as cross-reactive antibodies cannot be detected.

## Conclusions

Despite the limitations of a small sample size, this prospective study provided new insights on processes associated with favourable disease outcomes and protection from severe dengue upon a second encounter with DENV. Thus, single cell cytometry approaches combined with molecular profiling like the one presented here could be useful to help identify correlates of protection and define disease-protecting CD4^+^ and CD8^+^ T cell phenotypes that emerging anti-DENV vaccines under efficacy testing could aim to reproduce. The results also provide proof of concept for the potential of systems immunology approaches to identify discrete populations in the blood associated with increased odds of DHF, encouraging more granular examination of these cell compartments to identify specific biomarkers, useful for the design of diagnostic tools to predict disease outcomes of patients presenting with dengue fever at point of care.

## Supplementary information

**Additional file 1**: **TableS1: **CyTOF antibodies. Antibodies used for deep immunophenotyping in the study.

**Additional file 2**: **TableS2: **Cell surface makers included as parameters in viSNE analysis. Surface markers used for unsupervised high-dimensional analysis of major blood cell populations.

**Additional file 3**: **Fig.S1.** Gating strategy to define major memory T cell, MBC, monocyte and NK cell populations. **Fig. S2. **Relative percentage of populations differentially abundant between DF and DHF cases identified by CITRUS in dengue-naive healthy controls. **Fig. S3. **Comparison of transcriptional profiles of DF and DHF cases relative to transcription levels of uninfected healthy controls. **Fig. S4. **Comparison of transcriptional profiles of DF and DHF cases with two gene signatures predictive of progression to severe dengue. **Fig. S5. **Correlation analysis of a 10-gene set predictive of severe dengue with clinical parameters, chemokine levels and cellular signatures found in dengue cases. **Fig. S6. **Primary dengue study cohort characteristics. **Fig.S7.** Monocyte and NK cell sub-populations induced in DENV-infected individuals progressing to DF or DHF.

## Data Availability

Processed bulk RNA-seq data generated for this study are available as GEO series (GSE215835). Raw data are available upon request, subject to approval by our Institutional Data Access Committee (dataaccess@wehi.edu.au) to ensure preservation of patient confidentiality.

## Abbreviations

DF: Dengue fever
DHF: Dengue hemorrhagic fever
PBMC: Peripheral blood mononuclear cell
DALY: Disability-adjusted life years
DENV: Dengue virus
FcγR: Fc-gamma-receptor
ADE: Antibody dependent enhancement
ICOS: Inducible T cell co-stimulator
PD-1: Programmed cell death protein 1
CyTOF: Cytometry by time of flight
tSNE: t-distributed stochastic neighbour embedding
SOMs: Self-organizing maps
CITRUS: Cluster identification, characterization, and regression
Th cell: T helper cell
Tfh cell: T follicular helper cell
MBC: Memory B cell
NK cell: Natural killer cell
RNA-seq: RNA-sequencing
BTMs: Blood transcriptional modules
IPA: Ingenuity pathway analysis
GO: Gene ontology
KEGG: Kyoto Encyclopedia of Genes and Genomes
GBP: Guanylate-binding protein
TLR3: Toll like receptor 3
MMP: Metalloproteinase
RICTOR: Rapamycin-insensitive companion of mTOR
LARP1: La-related protein 1
NONO: Non-POU domain-containing octamer-binding protein
IFNAR: Interferon-alpha receptor
