# Supplementary material for: Integrated systems immunology approach identifies impaired effector T cell memory responses as a feature of progression to severe dengue fever
Source: J Biomed Sci. 2023 Apr 13;30:24. doi: 10.1186/s12929-023-00916-4 (PMC10103532; doi:10.1186/s12929-023-00916-4)
Supplement: Supplementary file 1 — Additional file 1: TableS1: CyTOF antibodies. Antibodies used for deep immunophenotyping in the study. [file 12929_2023_916_MOESM1_ESM.pdf]

Table S1: CyTOF antibodies

Antibody	Clone	Supplier
141Pr-conjugated anti-CCR6	11A9	Fluidigm
143Nd-conjugated anti-CD45RA	HI100	Fluidigm
146Nd-conjugated anti-IgD	IA6-2	Fluidigm
147Sm-conjugted anti-CD20	2H7	Fluidigm
149Sm-conjugated anti-CD25	2A3	Fluidigm
151Eu-conjugated anti-ICOS	DX29	Fluidigm
152Sm-conjugated anti-CD21	BL13	Fluidigm
153Eu-conjugated anti-CXCR5	RF8B2	Fluidigm
155Gd-conjugated anti-CD56	B159	Fluidigm
156Gd-conjugated anti-CXCR3	G025H7	Fluidigm
158Gd-conjugated anti-CD10	HI10a	Fluidigm
159Tb-conjugated anti-CCR7	G043H7	Fluidigm
160Gd-conjugated anti-CD14	RMO52	Fluidigm
165Ho-conjugated anti-CD19	HIB19	Fluidigm
167Er-conjugated anti-CD27	L128	Fluidigm
168Er-conjugated anti-CD8	SK1	Fluidigm
170Er-conjugated anti-CD3	UCHT1	Fluidigm
172Yb-conjugated anti-IgM	MHM-88	Fluidigm
174Yb-conjugated anti-CD4	SK3	Fluidigm
175Lu-conjugated anti-PD-1	EH12.2H7	Fluidigm
176Yb-conjugated anti-CD127	A019D5	Fluidigm
209Bi-conjugated anti-CD16	38G	Fluidigm
