# Supplementary material for: Integrated systems immunology approach identifies impaired effector T cell memory responses as a feature of progression to severe dengue fever
Source: J Biomed Sci. 2023 Apr 13;30:24. doi: 10.1186/s12929-023-00916-4 (PMC10103532; doi:10.1186/s12929-023-00916-4)
Supplement: Supplementary file 2 — Additional file 2: TableS2: Cell surface makers included as parameters in viSNE analysis. Surface markers used for unsupervised high-dimensional analysis of major blood cell populations. [file 12929_2023_916_MOESM2_ESM.pdf]

Table S2. Cell surface makers included as parameters in viSNE analysis

Cell population	viSNE parameters
Total MBCs	CD21, CD27, IgD, IgM, CCR6, CCR7, CXCR3, CXCR5 and CD45RA
T_{H1} memory $CD4^+$ T cells	CXCR5, CCR7, ICOS, PD-1, CD27, CD25 and CD127
T_{H2} memory $CD4^+$ T cells	CXCR5, CCR7, ICOS, PD-1, CD27, CD25 and CD127
T_{H17} memory $CD4^+$ T cells	CXCR5, CCR7, ICOS, PD-1, CD27, CD25 and CD127
Memory T_{FH} cells	CXCR3, CCR6, CCR7, ICOS, PD-1, CD27, CD25 and CD127
Memory $CD8^+$ T cells	CXCR3, CCR7, ICOS, PD-1, CD15, CD16, CD27, CD25 and CD127
Monocytes	CXCR3, CD4, CD8, CD10, CD14, CD16, CD27, CD45RA, CD56 and CD127
NK cells	CD4, CD8, CD16, CD25, CD27, CD45RA, CD127, ICOS and PD-1
