# Supplementary material for: Integrated systems immunology approach identifies impaired effector T cell memory responses as a feature of progression to severe dengue fever
Source: J Biomed Sci. 2023 Apr 13;30:24. doi: 10.1186/s12929-023-00916-4 (PMC10103532; doi:10.1186/s12929-023-00916-4)
Supplement: Supplementary file 3 — Additional file 3: Fig.S1. Gating strategy to define major memory T cell, MBC, monocyte and NK cell populations. Fig. S2. Relative percentage of populations differentially abundant between DF and DHF cases identified by CITRUS in dengue-naive healthy controls. Fig. S3. Comparison of transcriptional profiles of DF and DHF cases relative to transcription levels of uninfected healthy controls. Fig. S4. Comparison of transcriptional profiles of DF and DHF cases with two gene signatures predictive of progression to severe dengue. Fig. S5. Correlation analysis of a 10-gene set predictive of severe dengue with clinical parameters, chemokine levels and cellular signatures found in dengue cases. Fig. S6. Primary dengue study cohort characteristics. Fig.S7. Monocyte and NK cell sub-populations induced in DENV-infected individuals progressing to DF or DHF. [file 12929_2023_916_MOESM3_ESM.pdf]

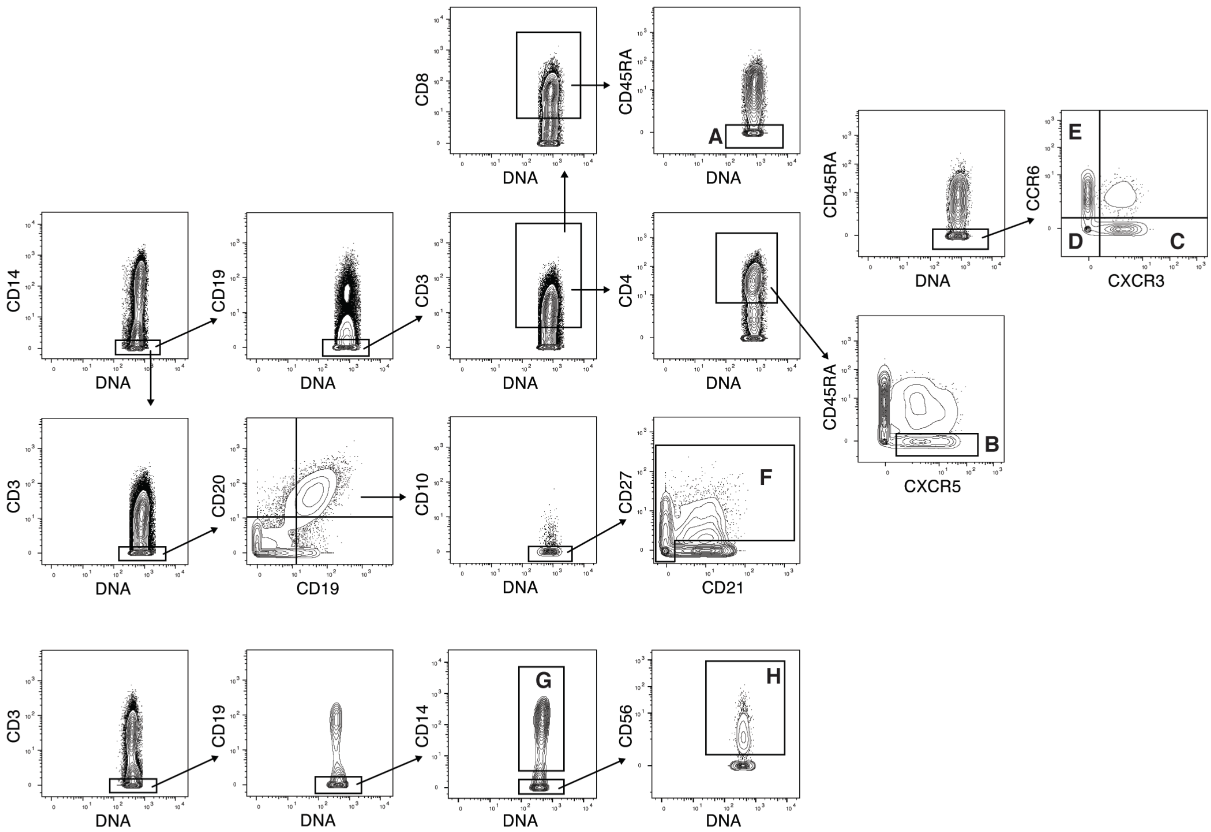

Fig. S1. Gating strategy to define major memory T cell, MBC, monocyte and NK cell populations. **A-H**. PBMCs collected at first presentation from DENV-positive individuals progressing to DF (n=6) or DHF (n=6) were stained with a panel of metal-labelled antibodies and analysed by CyTOF. Manual gating was used to select the following populations before FlowSOM clustering:

A. Memory $CD8^{+}$ T cells ($CD19^{-}CD3^{+}CD8^{+}CD45RA^{-}$),

B. Circulating memory T_{FH} cells ($CD19^{-}CD3^{+}CD4^{+}CD45RA^{-}CXCR5^{+}$),

C. T_{H1} memory $CD4^{+}$ T cells ($CD19^{-}CD3^{+}CD4^{+}CD45RA^{-}CCR6^{-}CXCR3^{+}$),

D. T_{H2} memory $CD4^{+}$ T cells ($CD19^{-}CD3^{+}CD4^{+}CD45RA^{-}CCR6^{-}CXCR3^{-}$),

E. T_{H17} memory $CD4^{+}$ T cells ($CD19^{-}CD3^{+}CD4^{+}CD45RA^{-}CCR6^{+}CXCR3^{-}$)

F. MBCs ($CD3^{-}CD19^{+}CD20^{+}CD10^{-}$)

G. Monocytes ($CD3^{-}CD19^{-}CD14^{+}$)

H. NK cells ($CD3^{-}CD19^{-}CD14^{-}CD56^{+}$)

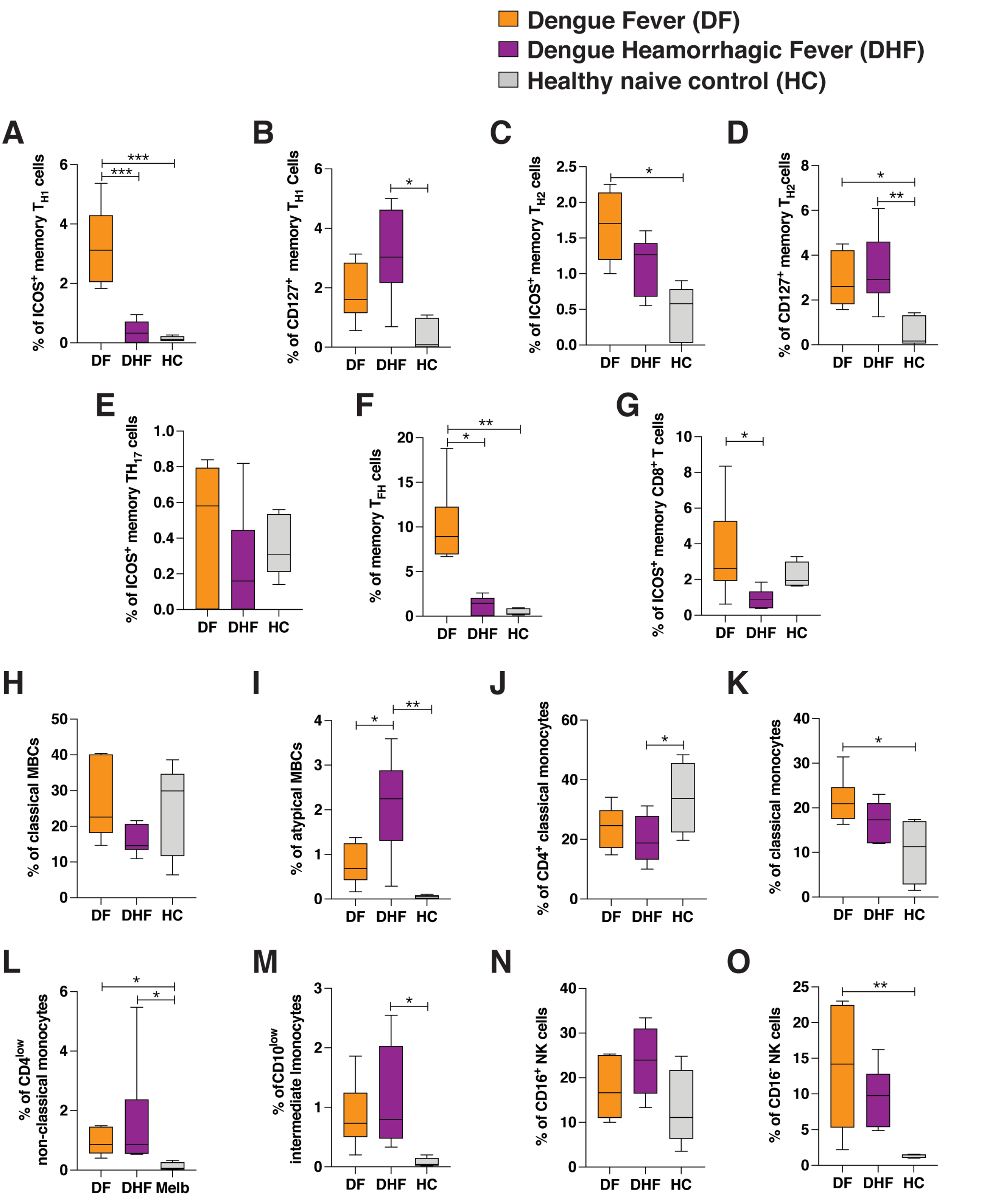

Fig. S2. Relative percentage of populations differentially abundant between DF and DHF cases identified by CITRUS in dengue-naïve healthy controls. **A-O**. PBMCs collected at first presentation from DENV-positive individuals progressing to DF (n=6), DHF (n=6) and healthy denge-naive controls (HC) from Melbourne, Australia (n=6) were stained with a panel of metal-labelled antibodies and analysed by CyTOF. Manual gating was used to identify

populations differentially abundant between DF and DHF cases found by CITRUS analysis in Figure 3:

A. ICOS$^+$ memory T$_{H1}$ cells (CD19$^-$CD3$^+$CD4$^+$CD45RA$^-$CCR6$^-$CXCR3$^+$ICOS$^+$CD27$^+$PD-1$^+$CCR7$^-$CD127$^-$)

B. CD127$^+$ memory T$_{H1}$ cells (CD19$^-$CD3$^+$CD4$^+$CD45RA$^-$CCR6$^-$CXCR3$^+$ICOS$^-$ PD-1$^-$CD27$^-$CCR7$^+$CD127$^+$)

C. ICOS$^+$ memory T$_{H2}$ cells (CD19$^-$CD3$^+$CD4$^+$CD45RA$^-$CCR6$^-$CXCR3$^-$ICOS$^+$CD27$^+$ PD-1$^+$CCR7$^-$CD127$^-$)

D. CD127$^+$ memory T$_{H2}$ cells (CD19$^-$CD3$^+$CD4$^+$CD45RA$^-$CCR6$^-$CXCR3$^-$ICOS$^-$CD27$^-$ PD-1$^-$CCR7$^+$CD127$^+$)

E. ICOS$^+$ memory T$_{H17}$ cells (CD19$^-$CD3$^+$CD4$^+$CD45RA$^-$CCR6$^+$CXCR3$^-$ICOS$^+$CD27$^+$PD-1$^+$CCR7$^-$CD127$^-$)

F. CXCR3$^+$ memory T$_{FH}$ cells (CD19$^-$CD3$^+$CD4$^+$CD45RA$^-$CXCR5$^+$CXCR3$^+$CCR6$^-$ICOS$^+$PD-1$^+$ CD27$^+$)

G. ICOS$^+$ memory CD8$^+$ T cells (CD19$^-$CD3$^+$CD8$^+$CD45RA$^-$CXCR3$^+$ICOS$^+$CD27$^+$PD-1$^+$CCR7$^-$CD127$^-$)

H. Class-switched classical MBCs (CD3$^-$CD19$^+$CD20$^+$CD10$^-$CD21$^+$CD27$^+$CXCR5$^+$CCR6$^+$ IgM$^-$IgD$^-$)

I. Class-switched atypical MBCs (CD3$^-$CD19$^+$CD20$^+$CD10$^-$CD21$^-$CD27$^-$CXCR5$^-$CCR6$^-$ IgM$^-$IgD$^-$)

J. CD4$^+$ classical monocytes (CD3$^-$CD19$^-$CD14$^+$CD16$^-$ CD4$^+$)

K. classical monocytes (CD3$^-$CD19$^-$CD14$^+$CD16$^-$)

L. CD4^{low} non-classical monocytes (CD3$^-$CD19$^-$CD14$^+$CD16$^+$CD4^{low})

M. CD10^{low} intermediate monocytes (CD3$^-$CD19$^-$CD14$^+$CD16^{low} CD10^{low})

N. CD16$^+$ NK cells (CD3$^-$CD19$^-$CD14$^-$CD56$^+$CD16$^+$)

O. CD16$^-$ NK cells (CD3$^-$CD19$^-$CD14$^-$CD56$^+$CD16$^-$)

Boxes represent the 25th to 75th percentiles, whiskers show the range (minimum to maximum), and lines represent the median of 6 biological replicates. Significance was determined by one-way ANOVA, *$p<0.05$, **$p<0.01$, ***$p<0.005$.

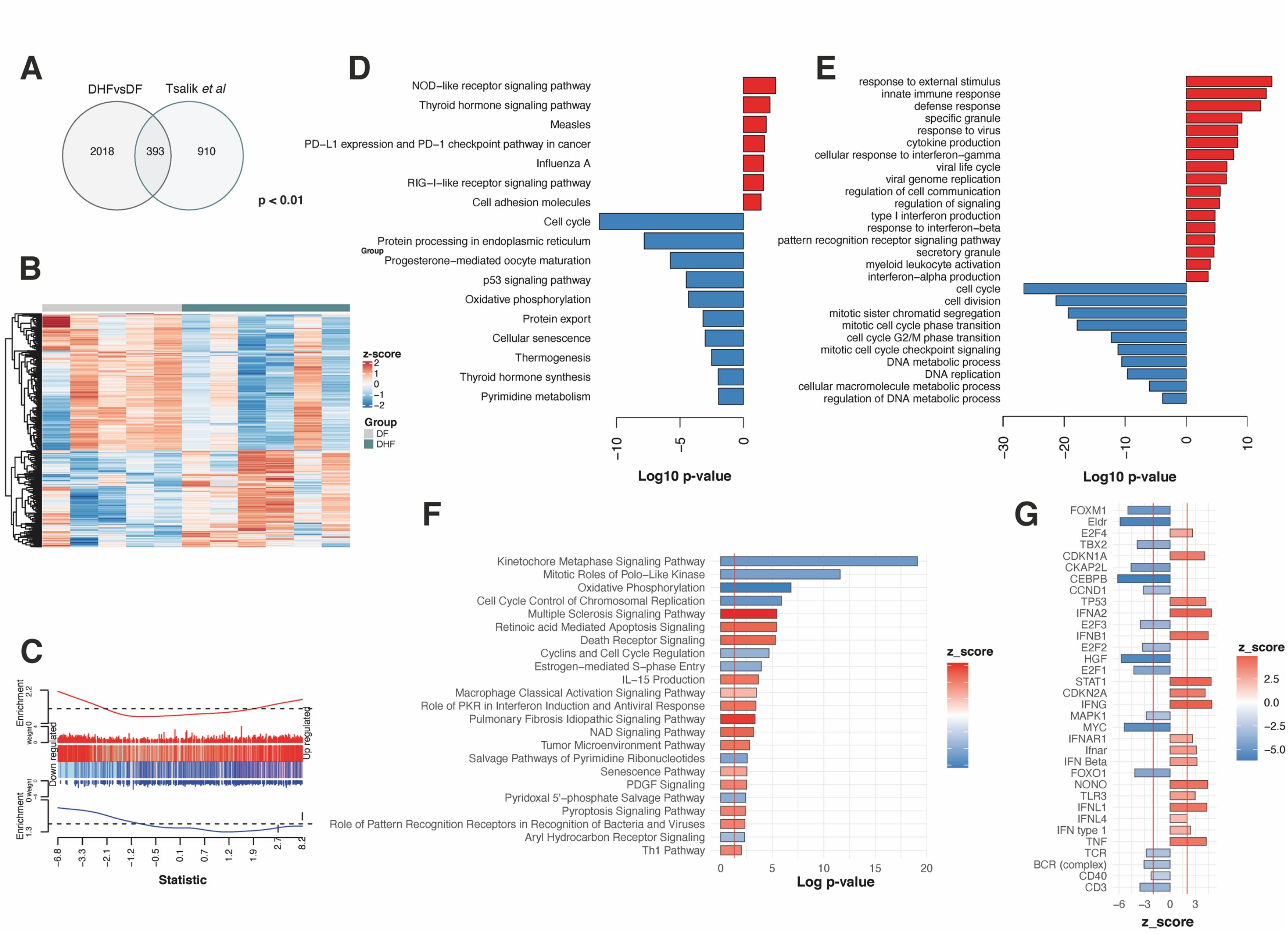

Fig. S3. Comparison of transcriptional profiles of DF and DHF cases relative to transcription levels of uninfected healthy controls. **A-F**. PBMCs collected at first presentation from DENV-positive individuals progressing to DF (n=5) or DHF (n=6) selected for RNA-seq analysis. **A**. Venn diagram showing overlap of differentially expressed genes between DF and DHF detected by RNA-seq and a transcriptional signature that defines genes differentially expressed in response to unclassified dengue relative to uninfected healthy controls [50] **B**. Hierarchical clustering heatmap displaying genes identified in *A*. **C**. Barcode plot representing gene set enrichment of genes identified in **A**, showing significant enrichment of genes upregulated in response to infection. Significance was tested using the ROAST test in limma. **D**. Bar plots showing significantly enriched GO terms using the 393 overlapping genes identified in **A**, scaled by Log10(P-value). Red GO terms are upregulated and blue GO terms are downregulated in DHF compared to DF. **E**. Bar plots showing significantly enriched KEGG pathways using the 393 overlapping genes identified in **A**, scaled by Log10(P-value). Red

KEGG pathways are upregulated and blue KEGG pathways are downregulated in DHF compared to DF. **F**. IPA canonical pathways significantly overrepresented in the 393 overlapping genes identified in **A** scaled by Log10(P-value). Pathways with a positive z-score in red are activated in DHF relative to DF, and pathways with a negative z-score in blue are inhibited in DHF compared to DF. The red line corresponds to a P value of 0.05. **G**. Upstream regulator analysis of the using the 393 overlapping genes identified in A. The red lines represent a significant activation z-score of ±2.

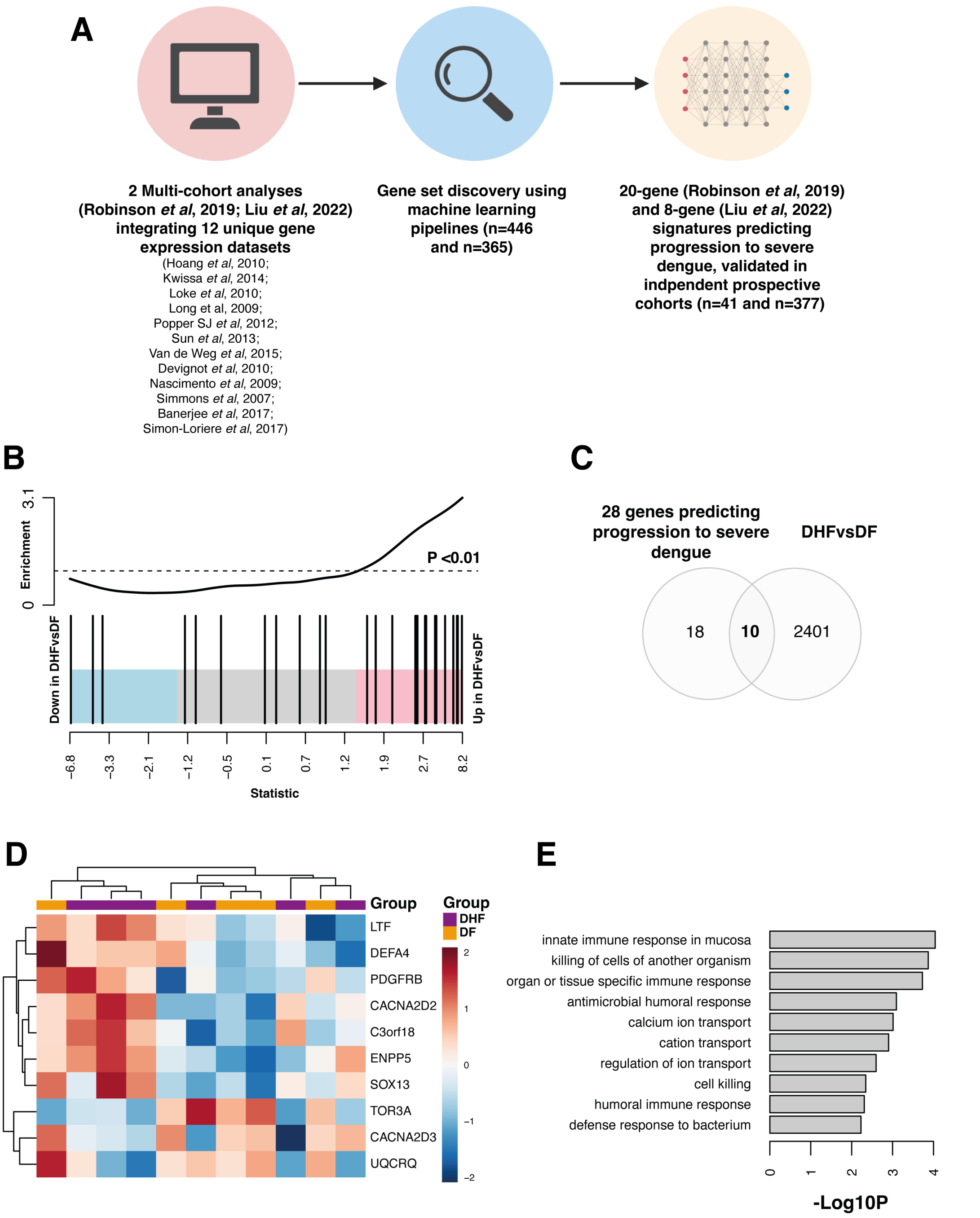

Fig. S4. Comparison of transcriptional profiles of DF and DHF cases with two gene signatures predictive of progression to severe dengue. **A**. Two multi-cohort analyses [51, 52] that together integrated 12 gene expression datasets identified two gene signatures, which predict progression to DHF. Signatures were validated in independent cohorts. These two gene signatures including 28 genes, were compared with DEG between DF and DHF detected by RNA-seq. **B**. Gene set enrichment test, showing significant enrichment of genes upregulated

in response to DHF. Significance was tested using ROAST Test in limma. **C**. Venn diagram showing overlap of DEGs between DF and DHF and the 28 genes present in the severe dengue predictive signature.**D**. Hierarchical clustering heatmap displaying genes identified in **B**. **E**. Bar plots showing significantly enriched GO terms using the 10 overlapping genes identified in **B**, scaled by -Log10(P-value).

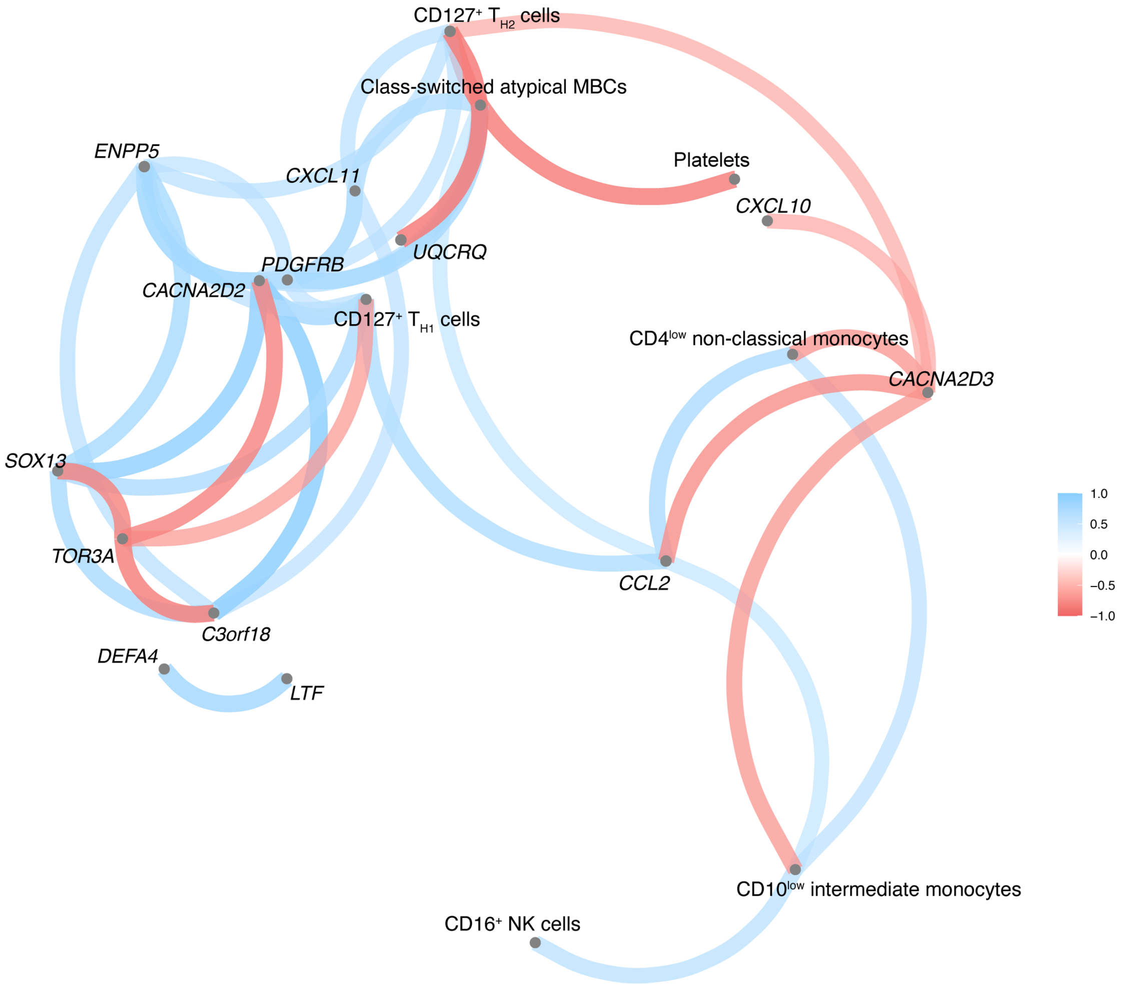

Fig. S5. Correlation analysis of a 10-gene set predictive of severe dengue with clinical parameters, chemokine levels and cellular signatures found in dengue cases. The relationship between, platelet counts, chemokine responses, cell populations significantly abundant in DHF cases identified by CITRUS and a 10-gene set identified by comparison of DEG between DF and DHF with validated gene signatures predictive of severe dengue [51, 52] was examined using Spearman correlation networks. Blue lines represent positive correlations, and red lines represent negative correlations. The distance between variables is determined by multidimensional scaling and represents the strength of the correlation, where highly correlated variables are positioned closer together.

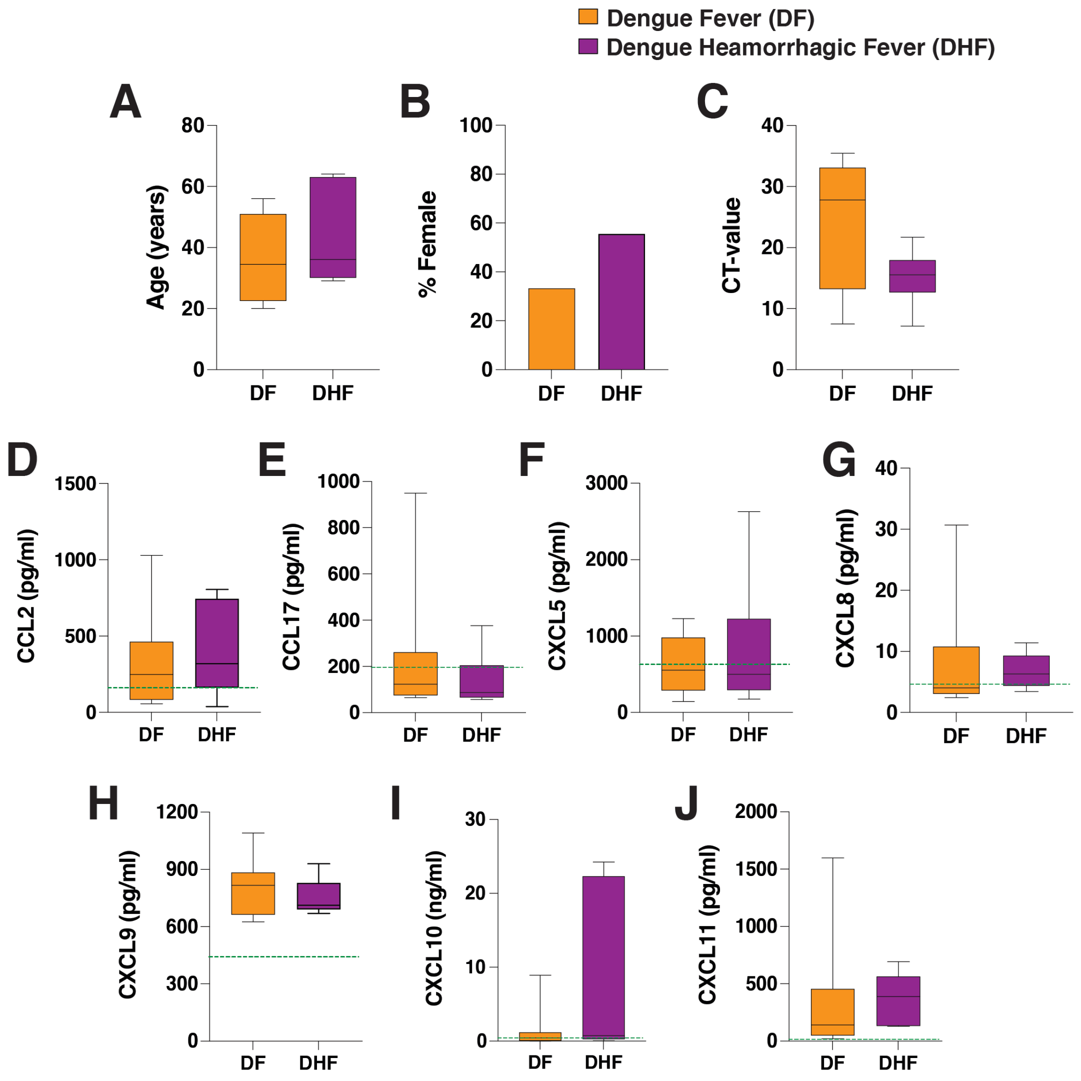

Figure S6. Primary dengue study cohort characteristics. **A-J**. Individuals with a confirmed primary DENV infection were recruited for the study at first presentation and followed up for progression to DF (n=8) or DHF (n=6). **A-C**. Clinical parameters determined in the study include age (**A**), gender (**B**) and CT-value (**C**). Boxes represent the 25th to 75th percentiles, whiskers show the range (minimum to maximum), and lines represent the median of 8 (DF) and 6 (DHF) biological replicates. Line plots depict mean $\pm$ SEM. Significance was determined by the Kruskal-Wallis test (**A**), the Chi-square test (**B**), and the Mann-Whitney test (**C**), *$p<0.05$. **D-J**. Mean chemokine levels. CCL2 (**D**), CCL17 (**E**), CXCL5 (**F**), CXCL8 (**G**), CXCL9 (**H**), CXCL10 (**I**) and CXCL11 (**J**) were determined in plasma samples of study participants. Boxes represent the 25th to 75th percentiles, whiskers show the range (minimum to maximum), and lines represent the median of 8 (DF) and 6 (DHF) samples. The dotted line depicts the average antibody background levels of uninfected healthy controls. Significance was determined by the Mann-Whitney test.

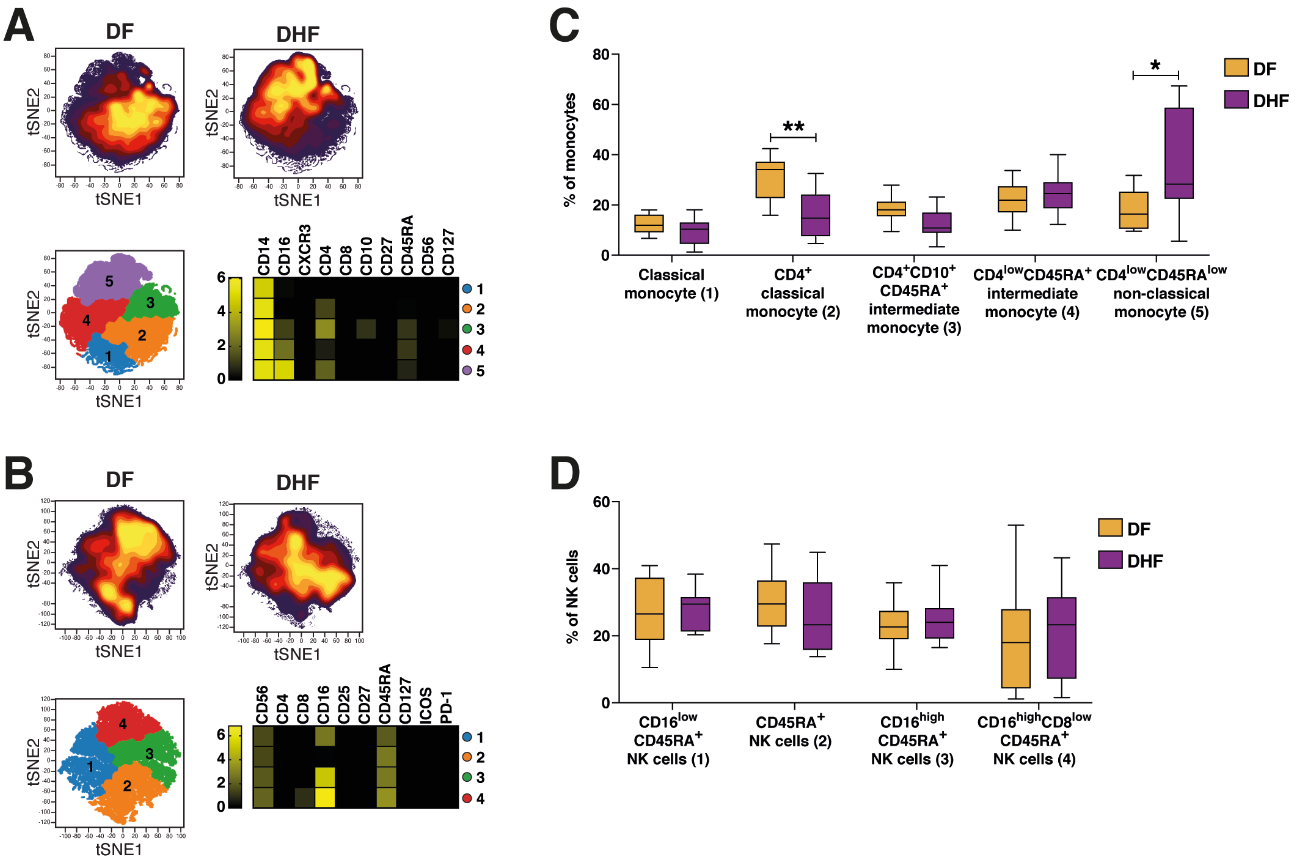

Fig. S7. Monocyte and NK cell sub-populations induced in DENV-infected individuals progressing to DF or DHF. **A-D**. PBMCs collected at first presentation from DENV-positive individuals progressing to DF (n=5) or DHF (n=4) with a confirmed either primary or secondary infection were stained with a panel of metal-labelled antibodies and analysed by CyTOF. tSNE analysis was performed and FlowSOM clustering was used to identify individual cell sub-populations within gated: **A**. Monocytes ($CD3^{-}CD19^{-}CD14^{+}$), **B**. NK cells ($CD3^{-}CD19^{-}CD14^{-}CD56^{+}$). The tSNE plots in the top panel display cell density and represent the pooled data for each group, while the lower panel shows a projection of the FlowSOM clusters on a tSNE plot. Heatmaps show the median marker expression for each FlowSOM cluster.**C, D**. Percentages of monocyte (**C**) and NK cell (**D**) subpopulations identified by FlowSOM clustering analysis in DF and DHF cases. Boxes represent the 25th-75th percentile, whiskers show the range (minimum to maximum) and lines represent the median of 11 (DF) and 10 (DHF) samples. Significance was determined by the Mann-Whitney test, *$p<0.05$ **$p<0.01$.
